# Synthesis, Characterization, and Anticancer Activity of New N,N′-Diarylthiourea Derivative against Breast Cancer Cells

**DOI:** 10.3390/molecules28176420

**Published:** 2023-09-03

**Authors:** Mohamed A. El-Atawy, Mai S. Alsubaie, Mohammed L. Alazmi, Ezzat A. Hamed, Demiana H. Hanna, Hoda A. Ahmed, Alaa Z. Omar

**Affiliations:** 1 Chemistry Department, Faculty of Science, Alexandria University, P.O. Box 426 Ibrahemia, Alexandria 21321, Egypt; mohamed.elatawi@alexu.edu.eg (M.A.E.-A.); alaazaki@alexu.edu.eg (A.Z.O.); 2 Chemistry Department, Faculty of Science, Taibah University, Yanbu 46423, Saudi Arabia; 3 Department of Chemistry, Faculty of Science, Cairo University, Giza 12613, Egypt; ahoda@sci.cu.edu.eg

**Keywords:** urea, thiourea, cytotoxicity, apoptosis, breast cancer

## Abstract

The goal of the current study was to prepare two new homologous series of N,N′-diarylurea and N,N′-diarylthiourea derivatives to investigate the therapeutic effects of these derivatives on the methodologies of inhibition directed on human MCF-7 cancer cells. The molecular structures of the prepared derivatives were successfully revealed through elemental analyses, ^1^H-NMR, ^13^C-NMR and FT-IR spectroscopy. The cytotoxic results showed that Diarylthiourea (compound **4**) was the most effective in suppressing MCF-7 cell growth when compared to all other prepared derivatives, with the most effective IC_50_ value (338.33 ± 1.52 µM) after an incubation period of 24 h and no cytotoxic effects on normal human lung cells (wi38 cells). Using the annexin V/PI and comet tests, respectively, treated MCF-7 cells with this IC_50_ value of the Diarylthiourea **4** compound displayed a considerable increase in early and late apoptotic cells, as well as an intense comet nucleus in comparison to control cells. An arrest of the cell cycle in the S phase was observed via flow cytometry in MCF-7 cells treated with the Diarylthiourea **4** compound, suggesting the onset of apoptosis. Additionally, ELISA research showed that caspase-3 was upregulated in MCF-7 cells treated with compound **4** compared to control cells, suggesting that DNA damage induced by compound **4** may initiate an intrinsic apoptotic pathway and activate caspase-3. These results contributed to recognizing that the successfully prepared Diarylthiourea **4** compound inhibited the proliferation of MCF-7 cancer cells by arresting the S cell cycle and caspase-3 activation via an intrinsic apoptotic route. These results, however, need to be verified through in vivo studies utilizing an animal model.

## 1. Introduction

Worldwide, breast cancer (BC) has an incidence rate of 11.7% (2,261,419 new cases), just slightly greater than the prevalence of lung cancer (11.4%) [1]. Among all cancers, breast cancer is one of the most common, representing 32% of cases in the latest information available from Egypt’s NCRP, the National Cancer Registry Programme [2]. Despite high survival rates in many developed nations, recent data show that Egypt’s has remained dismal, especially when measured over a period of five years [3]. Treatment of patients with breast cancer is becoming more difficult due to a number of additional issues, such as the adverse effects of standard therapies, including radiotherapy and chemotherapy [4]. Therefore, a great deal of research is being carried out currently, specifically to develop alternative strategies for treating breast cancer that can be employed as therapies, as an adjuvant treatment in addition to various treatments, or as chemopreventive medicines [5].

**Figure 1:**
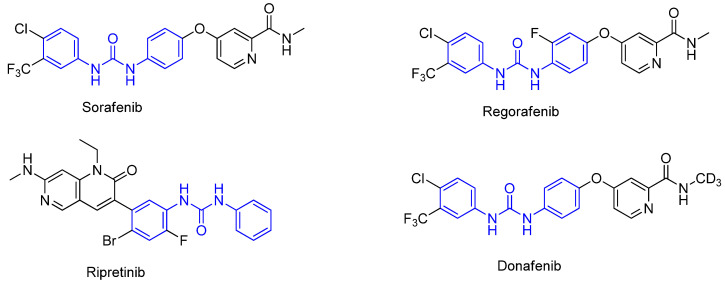
Some examples of anticancer agents containing diarylurea pharmacophore.

Diarylurea and diarylthiourea derivatives are privileged pharmacophores that are present in several pharmaceutically useful substances [6,7,8,9,10]. These pharmacophores have been the subject of intense interest due to their promising potential as anticancer agents [11,12,13,14,15,16], Figure 1. These compounds have shown promising results in preclinical studies and have the potential to be developed into effective cancer treatments. Urea derivatives are typically largely known for their antimicrobial properties [17,18,19]. However, research has shown that certain urea derivatives exhibit potent cytotoxic effects against cancer cells. By suppressing cell multiplication, promoting apoptosis (programmed cell death), and modifying cancer cell metabolism, these compounds can trigger cell death [13].

Thiourea derivatives, on the other hand, have demonstrated significant anticancer activity as well. These compounds have the potential to reverse medication resistance in cancer cells and have already been proven to suppress the proliferation of several different kinds of cancer cell lines [20,21,22,23,24,25]. Thiourea derivatives can target specific molecular pathways involved in cancer progression, such as inhibition of angiogenesis and modulation of cancer cell signaling pathways [26,27,28]. Furthermore, a recent study reported that sulfur-containing heterocyclic compounds have strong anti-proliferative effects on breast cancer cells [26,27,28].One key advantage of urea and thiourea derivatives as anticancer agents is their structural diversity, which permits the development of a variety of compounds with varying properties. This enables researchers to optimize their structures and tailor them for specific cancer types or drug delivery systems. Furthermore, many urea and thiourea derivatives have shown favorable pharmacokinetic properties, including good absorption, distribution, metabolism, and excretion profiles. This makes them suitable candidates for further development into drug candidates.

Herein, we aim to develop new diarylurea and diarylthiourea derivatives so that we may modulate the equilibrium between the lipophilicity and hydrophilicity of these compounds for the investigation of their inhibition mechanisms on human breast cancer cells. Therefore, our design for these new compounds involves introducing a long nonpolar alkyl terminal chain with between six and sixteen carbon atoms in order to enhance the permeability through lipophilic membranes. Moreover, introducing a fluorine group as a hydrogen bond acceptor group would improve the aqueous solubility of the designed compounds. On the one hand, various methods, including FT-IR, ^1^H-NMR, and ^13^C-NMR spectroscopy, were successful in illuminating the molecular structures of the produced derivatives. On the other hand, the efficiency of the most potent derivative (compound **4**), which showed the strongest IC_50_ value against human breast cancer, was evaluated using cytotoxic and apoptotic procedures.

## 2. Results and Discussion

### 2.1. Chemistry

**Scheme 1:**
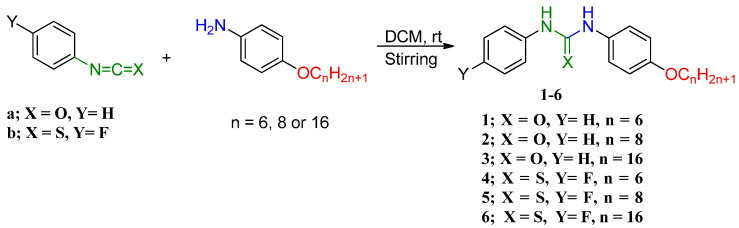
Synthesis of urea and thiourea derivatives **1**–**6**.

N,N′ disubstituted Urea and N,N′ disubstituted thiourea derivatives **1**–**6** were synthesized through direct reaction between equimolar amounts of either Phenylisocyanate or 4-fluorophenylisothiocyanate with alkoxy anilines, namely, 4-hexyloxyaniline, 4-octyloxyaniline or 4-hexadecyloxyaniline in dichloromethane at room temperature, as shown in Scheme 1. The infrared spectrum of compounds **1**–**6** displayed N-H stretching absorption peak in the region 3200–3320 cm^−1^. Moreover, the absorption due to the aliphatic C-H stretching appeared as absorption bands in the region 2920–2964 cm^−1^. Additionally compounds **1**–**3** exhibited sharp absorption peak in the region 1640–1650 cm^−1^ that corresponds to the carbonyl group stretching. The terminal alkoxy group protons could be detected in the proton NMR spectra with chemical shifts ranging from 0.82 to 3.99 ppm. In addition, the corresponding saturated aliphatic carbons of each of these compounds had signals in the carbon NMR spectrum within the range of 14–68 ppm. In addition, the benzene rings of compounds **1**–**6** have been detected utilizing NMR spectra of both protons and carbons. Accordingly, as a prototype, the aromatic protons of **4** appeared in proton NMR as two doublets, a doublet of doublets and a pseudo triplet (pst), and each of them represent two protons. The pair of doublets of equal area represent the four protons of the aryl ring bearing the alkoxy group. The doublet-of-doublet signal was observed for the two protons of the other fluorinated aromatic ring due to three-bond coupling with the neighboring aromatic proton (^3^*J*_H,H_ = 9.0 Hz) and four-bond coupling with the fluorine atom (^4^*J*_H,F_ = 5.0 Hz), and this peak has been ascribed for the two protons in *meta* position relative to the fluorine atom. The pseudo-triplet (pst) signal has been assigned to the two protons in *ortho* positions relative to the fluorine atom. The nearly equal three-bond coupling with nearby protons and fluorine atoms (^3^*J*_H,H_ ≈ ^3^*J*_H,F_ ≈ 8.8 Hz) may explain why the pst signal was observed rather than a doublet-of-doublet signal.

**Figure 2:**
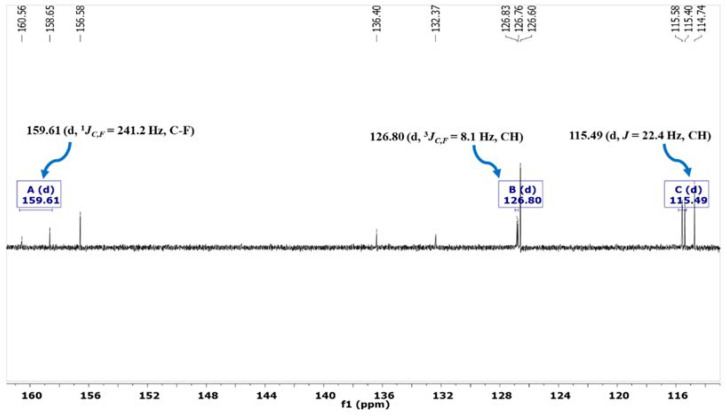
Carbon- fluorine splitting in Carbon-13 NMR spectrum of compound **4**.

The ^13^C-NMR spectrum, on the other hand, revealed downfield signals in the range of 114 to 160 ppm, which correspond to the aromatic ring carbons. Moreover, the carbon- fluorine splitting was detected in the Carbon-13 NMR spectra of compounds **4**–**6**. The coupling splitting that was seen in the Carbon-13 NMR spectra supports the presence of a fluorine atom in the 4-position relative to the thiourea moiety. As a prototype, ^13^C NMR spectrum of **4** exhibited a doublet signal at 159.61 ppm with one-bond ^19^F-^13^C coupling constant ^1^*J*_C,F_ ≈ 241.2 hertz, which was ascribed to the quaternary carbon directly bonded to the fluorine atom. Another ^19^F-^13^C splitting caused another doublet signal that appeared at ≈115 ppm with two-bond ^19^F-^13^C coupling constant ^2^*J*_C,F_ ≈ 22.4 hertz, which was attributed to the two *ortho* aromatic carbons relative to the fluorine atom. Furthermore, the hydrogenated carbon in *meta* position relative to the fluorine atom displayed a narrow doublet signal at ≈127 ppm due to three-bond ^19^F-^13^C coupling (^3^*J*_C,F_ ≈ 8.1 hertz), as shown in Figure 2.

### 2.2. Cytotoxicity Analyzes

**Figure 3:**
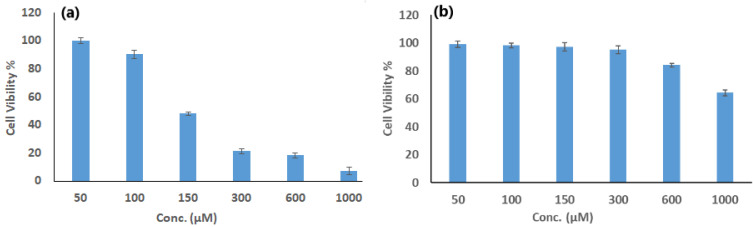
The correlation between the viability of MCF-7 cancer cells (**a**) and wi38 normal cells (**b**) following treatment with various doses of the compound **4** (50–1000 µM) for 24 h of incubation using MTT assay. The values are shown as mean ± SD of three separate analyses.

**Figure 4:**
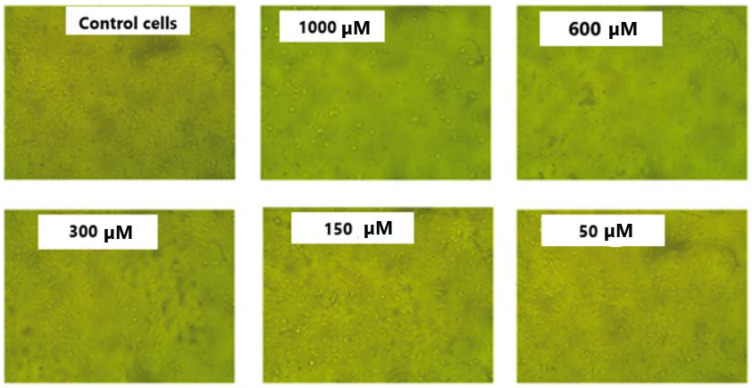
Microscopic observation of the morphological variations in MCF-7 cells treated for 24 h with varying doses (50–1000 µM) of the compound **4,** compared to untreated control cells.

The MTT, or 3-(4,5-dimethyl-2-thiazolyl)-2,5-diphenyl-2H-tetrazolium bromide, experiment was used to assess the toxic impact of the tested six compounds on the growth of MCF-7 cells. The percentage of viable MCF-7 cells for all the tested compounds at the different doses (50 to 1000 µM) for 24 h of incubation is displayed in Table S1, where compound **4** shows the highest toxic effect on cell viability with increasing doses up to 1000 µM (Figure 3a). The consequences of the obtained IC_50_ concentration, the required concentration of the tested compounds for a 50% inhibition of viability, of compounds **1**–**6** that were deduced from the relationship between the compound doses and their effects on MCF-7 cell viability, were 726.61 ± 1.29, 527.21 ± 1.28, 619.68 ± 4.33, 338.33 ± 1.52, 567.83 ± 4.28, and 711.88 ± 2.5 µM, respectively. This result suggests that compound **4**, with its lower IC_50_ concentration (338.3 ± 1.52 µM), may have greater potential anticancer properties. Similarly, previous research mentioned that the IC_50_ concentration (225 µM) of 4-nitrobenzoyl-3-allylthiourea showed an efficacy against the viability of breast cancer cells [29]. In contrast to untreated control cells, the treated MCF-7 with compound **4** had changes in cell size and shape, as well as a gradual decrease in cell viability with increasing concentrations of compound **4** (Figure 4).

The activity of the LDH, lactate dehydrogenase, enzyme was checked experimentally to offer extra confirmation of compound **4**’s inhibition of the growth of MCF-7 cells. The obtained results showed that the LDH levels in the compound **4**-treated MCF-7 cells increased significantly (521.77 ± 30.8 U/L), compared with their levels in untreated control cells (85.35 ± 4.2 U/L), *p* < 0.001. Thus, the obtained findings of MTT and LDH assays suggested the potent efficiency of compound **4** against the proliferation of tested MCF-7 cells. In addition, compound **4** demonstrated a markedly elevated percentage of cell viability in normal human lung cells, wi38 cells, with an elevated IC_50_ value (1463.18 ± 4.78 µM) after being cultured with compound **4** at doses ranging from 50 to 1000 µM; the obtained outcomes are shown in Figure 3b. These findings disclosed that compound **4** exhibited selective toxicity on cancer cells, whereas it displayed safety and biocompatibility characteristics in relation to healthy cells.

### 2.3. Estimation of Apoptosis and DNA Damage Generation in MCF-7 Cells

**Figure 5:**
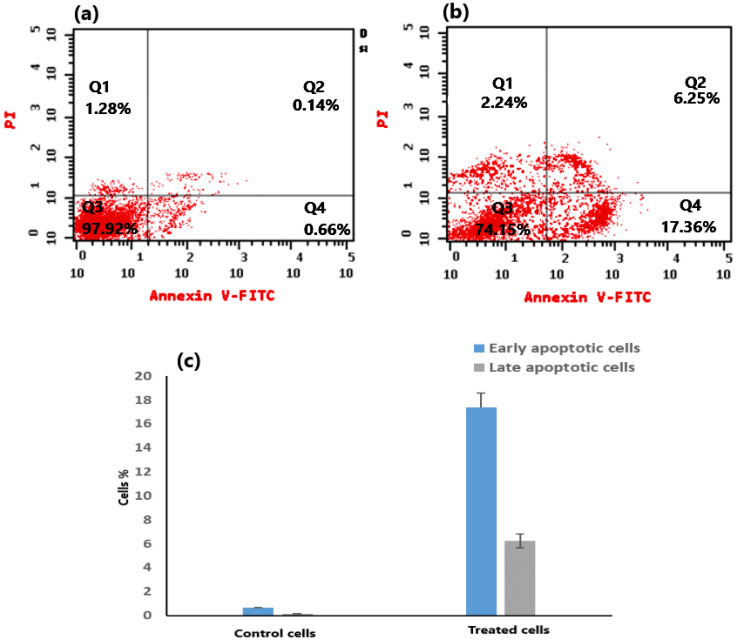
The apoptotic profile of MCF-7 cells was recorded by flow cytometry, revealing the following subpopulations relative to untreated control cells (**a**): Q1 represents necrotic cells (An−, PI+), Q2 represents late apoptotic cells (An+, PI+), Q3 represents viable cells (An−, PI−), and Q4 represents early apoptotic cells (An+, PI−) for the tested compound **4** (**b**) in comparison with untreated control cells. The histogram exhibits the proportion of both early and late apoptotic cells in compound **4**-treated cells versus untreated cells (**c**). Data from three separate analyses are shown as mean ± SD, *p* < 0.001.

**Figure 6:**
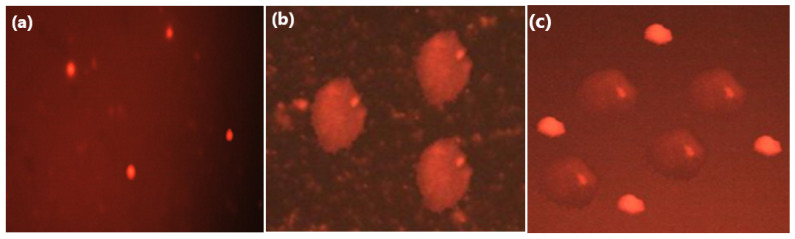
Fluorescence microscopy pictures exhibit that compound **4** generates a comet nucleus in both MCF-7 cells treated at its IC_50_ level (**b**), and MCF-7 cells treated at its IC_50_ level after treatment with the caspase-3 inhibitor Z-DEVD-FMK (**c**), in contrast to a normal nucleus in untreated MCF-7 cells (**a**).

- The ability of compound **4** to cause apoptosis and damage DNA in MCF-7 cells was tested with Annexin V/PI and comet analyses. Figure 5a,b illustrate the outcomes of flow cytometry analysis using annexin V/PI staining for cells treated with compound **4**, as compared to untreated cells. The proportion of apoptotic cells (including both early and late stages) was found to be significantly higher in the compound **4**-treated MCF-7 cells in comparison to untreated ones. Moreover, the % of both early and late apoptotic cells in compound **4**-treated cells in comparison to untreated ones is displayed in Figure 5c.
- The comet assay approach is also utilized for assessing the destruction of DNA generated in MCF-7 cells treated with compound **4** that was pretreated with a caspase-3 inhibitor (Z-DEVD-FMK). As shown in Table 1, there was a significant difference in the value of the Olive tail moment (OTM) between cells treated with compound **4**, as well as cells treated with compound **4** that have been previously treated with caspase-3 inhibitors, and the untreated control ones. Moreover, the obtained images using fluorescence microscopy (Figure 6) exhibited the fact that intact nuclei were observed in the untreated control cells (Figure 6a), whereas a comet-resembling structure was observed in both the cells treated with compound **4** (Figure 6b) and the cells treated with compound **4** that were pretreated with the inhibitor (Figure 6c), indicating that compound **4** induced DNA damage in the treated cells independently of caspase-3 activity. Based on these findings, it is possible that compound **4** effectively promotes the death of breast cancer cells by generating DNA damage, which subsequently initiates an intrinsic apoptosis pathway and consequently activates caspase-3.

### 2.4. Possible Apoptotic Pathways of Compound **4** in MCF-7 Cells

- Examining the Cell Cycle

**Figure 7:**
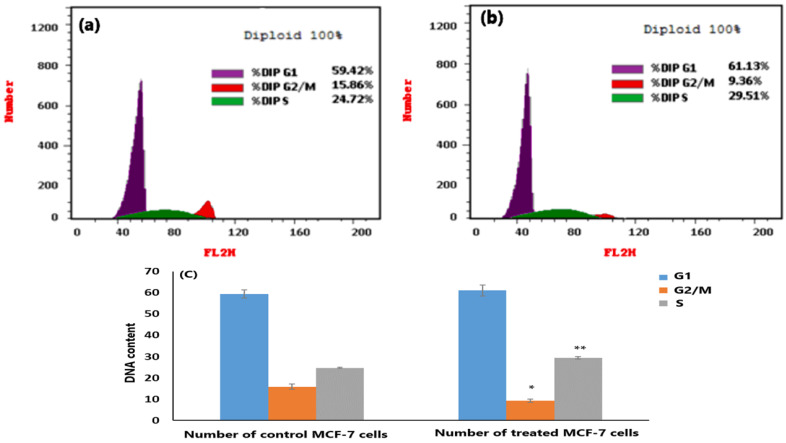
Multiple stages of the cell cycle were analyzed using flow cytometry in both untreated (**a**) and treated MCF-7 cells (**b**) at the IC_50_ level of compound **4** (338.33 ± 1.52 µM). The histogram shows the distribution of untreated and compound **4**-treated cells across the cell cycle (**c**). The findings are shown as mean ± SD of three consecutive assessments, (* *p* < 0.01 and ** *p* < 0.001).

One of the most important strategies for halting the expansion of cancer cells is to disrupt their normal cell cycle progression [30]. Thus, by using flow cytometry, it was probable to analyze the influence of compound **4** on the control of the MCF-7 cell cycle progression. As seen in Figure 7b, compound **4** generated a greater number of cells in the S phase in treated MCF-7 cells as compared to the MCF-7 control cells (Figure 7a), confirming the arresting of the cell cycle at the S phase that triggers apoptosis. It is possible that this occurred because of an interruption in DNA synthesis, which would have stopped the cell cycle and led to apoptosis. In addition, Figure 7c depicts a standard histogram comparing control and compound **4**-treated MCF-7 cells at each stage of the cell cycle.

A cell cycle stoppage at G1-S as well as G2-M transition may be due to damaging DNA or the influence of DNA damage upon CDK inhibitory proteins (P27 and P21) [31]. These kinds of proteins control the cell cycle by modulating the functions of complexes made up of cyclins and cyclin-dependent kinases (CDKs). For instance, CDK2/cyclin inhibition can trigger S-phase progression delays [30].

- Quantification analysis of the Caspase-3 ELISA assay

**Figure 8:**
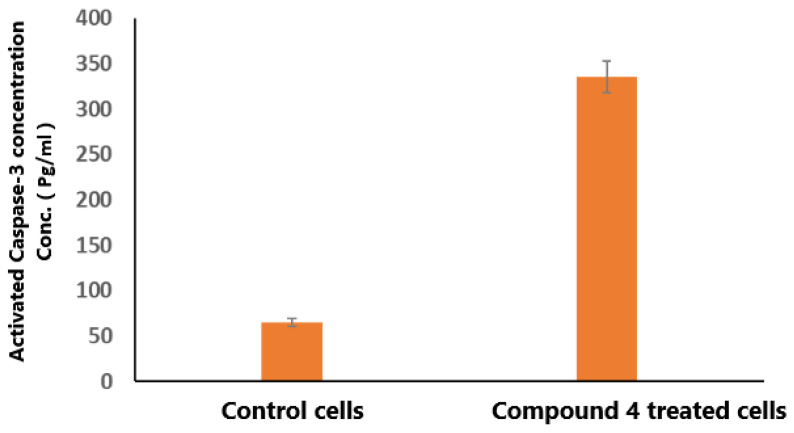
Comparison of cleaved caspase-3 protein levels in MCF-7 cells exposed to IC_50_ dose (338.33 ± 1.52 µM) of compound **4** with control cells employing ELISA approach. The findings are shown as mean ± SD of three consecutive assessments, *p* < 0.001.

In the present investigation, an ELISA technique was utilized to examine the levels of activated caspase-3 expression in MCF-7 cells that had been exposed to compound **4**. The obtained findings are displayed in Figure 8, which declares that the cleaved caspase-3 (activated caspase-3) levels in treated cells were significantly elevated compared to untreated cells (*p* < 0.001). Caspase-3 has been reported to be essential for DNA fragmentation and morphological alterations connected to apoptosis [32]. Therefore, the generation of DNA damage by compound **4** in MCF-7 cells (as validated by the comet assay in the presence of Z-DEVD-FMK as a caspase-3 inhibitor) may have triggered the intrinsic apoptotic pathway and resulted in caspase-3 activation.

## 3. **Experimental**

### 3.1. Instruments and Apparatus

The infrared spectra were recorded as potassium bromide (KBr) discs on a Perkin-Elemer FT-IR (Fourier-transform infrared spectroscopy). The nuclear magnetic resonance spectra were performed at ambient temperature (~25 °C) on a (JEOL) 400 MHz spectrophotometer. Elemental analyses were carried out utilizing a flash 2000 CHNS/O analyzer, Thermo Scientific (Waltham, MA, USA).

### 3.2. General Method for Synthesis of Diarylurea or Diarylthiourea Derivatives **1**–**6**

Phenyl isocyanate or 4-fluorophenyl isothiocyanate (1.00 mmol) was dissolved in 15 mL methylene chloride at room temperature. Then alkoxyaniline (1.01 mmol) was added gradually while stirring constantly, and the reaction progress was followed up by using TLC. Once a solid had formed after 40 min, it was filtered, washed with hexane, and then dried under vacuum. Recrystallization was carried out using hot ethanol as solvent.

1-(4-(hexyloxy)phenyl)-3-phenylurea **1**

Yield: 90%; IR (KBr): $\bar{v}$ 3316 (N-H), 3050 (S_P_^2^=C-H), 2925 (S_P_^3^ -C-H) and 1650 (C=O) cm^−1^. ^1^H NMR (DMSO-*d*_6_, 500 MHz): δ 8.58 (s, 1H, NH), 8.45 (s, 1H, NH), 7.40 (dd, *J* = 8.6, 1.0 Hz, 2H, Ar-H), 7.30 (d, *J* = 9.0 Hz, 2H, Ar-H), 7.22 (t, *J* = 7.9 Hz, 2H, Ar-H), 6.90 (t, *J* = 7.4 Hz, 1H, Ar-H), 6.80 (d, *J* = 8.9 Hz, 2H, Ar-H), 3.85 (t, *J* = 6.5 Hz, 2H, OCH_2_), 1.68–1.59 (m, 2H, CH_2_), 1.35 (m, 2H, CH_2_), 1.28–1.21 (m, 4H, 2CH_2_) and 0.87–0.77 (m, 3H, CH_3_) ppm. ^13^C NMR (DMSO-*d*_6_, 126 MHz): δ 154.37 (C=O), 150.50 (Ph-C), 140.45 (Ph-C), 129.27 (Ph-C), 122.08 (Ph-C), 120.48 (Ph-C), 118.55 (Ph-C), 115.45 (Ph-C), 115.05 (Ph-C), 68.05 (OCH_2_), 31.58 (CH_2_), 29.27 (CH_2_), 25.77 (CH_2_), 22.64 (CH_2_) and 14.47 (CH_3_) ppm. C_19_H_24_N_2_O_2_ requires: C, 73.03; H, 7.76; N, 8.97% found: C, 73.45; H, 7.85; N, 9.12%. MS M^+^ at *m*/*z* 312.29 (44%).

1-(4-(octyloxy)phenyl)-3-phenylurea **2**

Yield: 93%; IR (KBr): $\bar{v}$ 3290 (N-H), 3049 (S_P_^2^=C-H), 2924 (S_P_^3^ -C-H) and 1642 (C=O) cm^−1^. ^1^H NMR (DMSO-*d*_6_, 500 MHz): δ 8.71 (s, 1H, NH), 8.59 (s, 1H, NH), 7.40 (d, *J* = 7.9 Hz, 2H, Ar-H), 7.29 (d, *J* = 8.9 Hz, 2H, Ar-H), 7.22 (t, *J* = 7.9 Hz, 2H, Ar-H), 6.90 (t, *J* = 7.3 Hz, 1H, Ar-H), 6.80 (d, *J* = 9.0 Hz, 2H, Ar-H), 3.85 (t, *J* = 6.5 Hz, 2H, OCH_2_), 1.69–1.59 (m, 2H, CH_2_), 1.40–1.31 (m, 2H, CH_2_), 1.30–1.16 (m, 8H, 4CH_2_) and 0.82 (t, *J* = 6.9 Hz, 3H, CH_3_) ppm.^13^C NMR (DMSO-*d*_6_, 126 MHz): δ 154.32 (C=O), 153.30 (Ph-C), 140.52 (Ph-C), 133.23 (Ph-C), 129.26 (Ph-C), 122.03 (Ph-C), 120.42 (Ph-C), 118.52 (Ph-C), 115.07 (Ph-C), 68.05 (OCH_2_), 31.78 (CH_2_), 29.30 (CH_2_), 29.29 (CH_2_), 29.22 (CH_2_), 26.09 (CH_2_), 22.63 (CH_2_) and 14.51 (CH_3_) ppm. C_21_H_28_N_2_O_2_ requires: C, 74.07; H, 8.31; N, 8.23% found: C, 74.29; H, 8.42; N, 8.41%. MS M^+^ at *m*/*z* 340.34 (86%).

1-(4-(hexadecyloxy)phenyl)-3-phenylurea **3**

Yield: 89%; IR (KBr): $\bar{v}$ 3316 (N-H), 3057 (S_P_^2^=C-H), 2921 (S_P_^3^ -C-H) and 1639 (C=O) cm^−1^. ^1^H NMR (CDCl_3_, 500 MHz): δ 8.22 (s, 1H, NH), 8.20 (s, 1H, NH), 7.36–7.27 (m, 4H, Ar-H), 7.22 (d, *J* = 5.0 Hz, 2H, Ar-H), 7.11–7.06 (m, 1H, Ar-H), 6.88 (d, *J* = 7.7 Hz, 2H, Ar-H), 3.89 (t, *J* = 6.3 Hz, 2H, OCH_2_), 1.82–1.72 (m, 4H, 2CH_2_), 1.68–1.58 (m, 4H, 2CH_2_), 1.48– 1.38 (m, 4H, 2CH_2_), 1.34–1.21 (m, 16H, 8CH_2_) and 0.86 (t, *J* = 6.3 Hz, 3H, CH_3_) ppm. ^13^C NMR (CDCl_3_, 126 MHz): δ 154.78 (C=O), 151.96 (Ph-C), 142.59 (Ph-C), 133.22 (Ph-C), 128.87 (Ph-C), 122.15 (Ph-C), 120.01 (Ph-C), 118.33 (Ph-C), 114.99 (Ph-C), 68.32 (OCH_2_), 31.94 (CH_2_), 29.90 (CH_2_), 29.87 (CH_2_), 29.83 (CH_2_), 29.80 (CH_2_), 29.71 (CH_2_), 29.68 (CH_2_), 29.62 (CH_2_), 29.60 (CH_2_), 29.43 (CH_2_), 29.38 (CH_2_), 29.33 (CH_2_), 26.07 (CH_2_), 22.71 (CH_2_) and 14.14 (CH_3_) ppm. C_29_H_44_N_2_O_2_ requires: C, 76.93; H, 9.82; N, 6.12% found: C, 76.72; H, 9.94; N, 6.39%. MS M^+^ at *m*/*z* 452.79 (30%).

1-(4-fluorophenyl)-3-(4-(hexyloxy)phenyl)thiourea **4**

Yield: 92%; IR (KBr): $\bar{v}$ 3216 (N-H), 3019 (S_P_^2^=C-H), 2964 (S_P_^3^ -C-H) and 1553 (C=S) cm^−1^. ^1^H NMR (DMSO-*d*_6_, 500 MHz): δ 9.57 (s, 1H, NH), 9.53 (s, 1H, NH), 7.40 (dd, 2H, ^3^*J*_H,H_ = 9.0, ^4^*J*_H,F_ 5.0 Hz, Ar-H), 7.24 (d, 2H, *J* = 8.9 Hz, Ar-H), 7.11 (pst, 2H, ^3^*J*_H,H_ = 8.8, ^3^*J*_H,F_ 8.8 Hz, Ar-H), 6.85 (d, 2H, ^3^*J_H,H_* = 8.9 Hz, Ar-H), 3.90 (t, 2H, *J* = 6.5 Hz, OCH_2_), 1.71–1.55 (m, 2H, CH_2_), 1.44–1.33 (m, 2H, CH_2_), 1.31–1.22 (m, 4H, 2CH_2_) and 0.84 (t, *J* = 7.1 Hz, 3H, CH_3_) ppm. ^13^C NMR (DMSO-*d*_6_, 126 MHz): δ 180.70 (C=S), 159.61 (d, ^1^*J_C,F_* = 241.2 Hz, C-F), 156.58 (CH), 136.40 (C), 132.37 (C), 126.80 (d, ^3^*J_C,F_* = 8.1 Hz, CH), 126.60 (CH), 115.49 (d, ^2^*J_C,F_* = 22.4 Hz, CH), 114.74 (CH), 68.07 (OCH_2_), 31.54 (CH_2_), 29.19 (CH_2_), 25.75 (CH_2_), 22.63 (CH_2_) and 14.48 (CH_3_) ppm. C_19_H_23_FN_2_OS requires: C, 65.86; H, 6.70; N, 8.09% found: C, 65.74; H, 6.81; N, 8.27%. MS M^+^ at *m*/*z* 346.67 (64%).

1-(4-fluorophenyl)-3-(4-(octyloxy)phenyl)thiourea **5**

Yield: 91%; IR (KBr): $\bar{v}$ 3218 (N-H), 3021 (S_P_^2^=C-H), 2924 (S_P_^3^ -C-H) and 1544 (C=S) cm^−1^. ^1^H NMR (DMSO-*d*_6_, 500 MHz): δ 9.63 (s, 2H, 2NH), 7.41 (dd, 2H, ^3^*J*_H,H_ = 9.0, ^4^*J*_H,F_ 5.0 Hz, Ar-H), 7.25 (d, 2H, ^3^*J*_H,H_ = 8.9 Hz, Ar-H), 7.11 (pst, 2H, ^3^*J*_H,H_ = 8.8, ^3^*J*_H,F_ 8.8 Hz, Ar-H), 6.84 (d, 2H, ^3^*J*_H,H_ = 9.0 Hz, Ar-H), 3.89 (t, 2H, *J* = 6.5 Hz, OCH_2_), 1.73–1.59 (m, 2H, CH_2_), 1.43–1.32 (m, 2H, CH_2_), 1.32–1.16 (m, 8H, 4CH_2_) and 0.82 (t, 3H, *J* = 7.0 Hz, CH_3_) ppm.^13^C NMR (DMSO-*d*_6_, 126 MHz): δ 180.67 (C=S), 159.64 (d, ^1^*J_C,F_* = 243.7 Hz, C-F), 156.54 (C), 136.43 (C), 136.41 (C), 132.41 (C), 126.69 (d, ^3^*J_C,F_* = 8.1 Hz, CH), 126.52 (CH), 115.47 (d, ^2^*J_C,F_* = 22.5 Hz, CH), 114.72 (CH), 68.06 (OCH_2_), 31.79 (CH_2_), 29.30 (CH_2_), 29.28 (CH_2_), 29.23 (CH_2_), 26.08 (CH_2_), 22.63 (CH_2_) and 14.51 (CH_3_) ppm. C_21_H_27_FN_2_OS requires: C, 67.34; H, 7.28; N, 7.48% found: C, 67.21; H, 7.49; N, 7.64%. MS M^+^ at *m*/*z* 374.35 (44%).

1-(4-fluorophenyl)-3-(4-(hexadecyloxy)phenyl)thiourea **6**

Yield: 94%; IR (KBr): $\bar{v}$ 3223 (N-H), 3023 (S_P_^2^=C-H), 2920 (S_P_^3^ -C-H) and 1555 (C=S) cm^−1^. ^1^H NMR (CDCl_3_, 500 MHz): δ 9.25 (s, 2H, 2NH), 7.42–7.30 (m, 2H, Ar-H), 7.27–7.19 (m, 2H, Ar-H), 7.13–7.00 (m, 2H, Ar-H), 6.97–6.86 (m, 2H, Ar-H), 3.99–3.88 (m, 2H, OCH_2_), 1.85–1.67 (m, 2H, CH_2_), 1.44–1.38 (m, 2H, CH_2_), 1.37–1.18 (m, 24H, 12CH_2_) and 0.86 (t, 3H, *J* = 6.9 Hz, CH_3_) ppm. ^13^C NMR (CDCl_3_, 126 MHz): δ 181.41 (C=S), 156.49, 153.79, 127.45, 127.04, 123.54, 116.67, 115.36, 114.91, 68.47 (OCH_2_), 32.02 (CH_2_), 31.06 (CH_2_), 29.79 (CH_2_), 29.78 (CH_2_), 29.75 (CH_2_), 29.70 (CH_2_), 29.69 (CH_2_), 29.68 (CH_2_), 29.66 (CH_2_), 29.46 (CH_2_), 29.14 (CH_2_), 26.06 (CH_2_), 26.03 (CH_2_), 22.79 (CH_2_) and 14.24 (CH_3_) ppm. C_29_H_43_FN_2_OS requires: C, 71.55; H, 8.92; N, 5.76% found: C, 71.38; H, 8.79; N, 5.89%. MS M^+^ at *m*/*z* 486.30 (37%).

### 3.3. Cytotoxicity Assays

#### 3.3.1. Cell Line Preparation

The human cells that were utilized in the study included breast cancer cells, specifically the MCF-7 cell lines, along with normal lung cells, specifically the WI38 cell lines. These cell lines were taken from the American Type Culture Collection (ATCC). These cells were maintained in RPMI media with penicillin (100 U/mL), glutamine, 10% fetal bovine serum, and streptomycin (100 g/mL) in a humidified incubator (5% CO_2_, 37 °C).

#### 3.3.2. Cytotoxicity Test Using the MTT Approach

As indicated before [30,33], the prepared compounds (**1**–**6**) were tested for their capacity to inhibit the growth of MCF-7 cancer cells, employing the MTT experiment. In short, 1 × 10^5^ of MCF-7 cells/mL were seeded into each well of a 96-well plate and cultured in the growth mixture at 37 °C overnight. The MCF-7 cells were then exposed to the test compounds at 37 °C for 24 h at doses that ranged from 50 to 1000 µM.

The cells were then observed under an inverted microscope (Leica DMI3000B, Mannheim, Germany) for toxicity markers in both the control and treated cells. Following this, the cells were subjected to MTT solution, which caused MTT to be converted into formazan (MTT’s metabolic product), the amount of which was directly proportionate to the total number of viable cells. The formazan crystal was then extracted from the cells by treating them with dimethyl sulfoxide. Microplate reader (Bio-Rad, Hercules, CA, USA) readings were taken at 570 nm, and the percentage of live MCF-7 cells was calculated using the following Formula (1) [34,35] to indicate the influence of the studied compounds on the proliferation of the tested cells.

Cell viability % = (Abs. of tested compound − Abs. of blank)/(Abs. of control − Abs. of blank) × 100 (1)

The half-suppressing dose (IC_50_) of the tested compounds was estimated via a dose-response curve following incubation for 24 h. To further determine whether the compounds were safe for use on living tissues, the MTT technique was used at concentrations ranging from 50 to 1000 µM to analyze the influence of the compounds on the viability of healthy WI38 cells. Results are presented as the mean of three independent evaluations of each value.

#### 3.3.3. Measurement of Lactate Dehydrogenase (LDH) by Leakage Test

The levels of the released lactate dehydrogenase enzyme (LDH) in compound **4**-treated MCF-7 cells were detected using the double-antibody sandwich ELISA technique (SunRed, Cat. N. 201-28-0094) to further estimate the cytotoxic impact of the tested compound **4** against MCF-7 cells. In brief, the compound **4**-treated MCF-7 cancer cells were centrifuged, and 40 µL of the resulted supernatant was added to the tested wells. Following this, LDH-antibody (10 µL) and Streptavidin-HRP (50 µL) were transferred to each well for analysis. Subsequently, the 96-well plate was gently shaken, and incubated for 60 min at 37 °C. Following washing, chromogen solution A (50 µL) and chromogen solution B (50 µL) were transferred to each of the test wells, and the plates were left to incubate at 37 Celsius, out of the light, for 10 min. Next, each well was treated with stop solution (50 µL). Finally, the optical density, OD, for the tested plate was measured at 450 nm. Using the prepared standard curve, the concentration of the released LDH was estimated.

### 3.4. Apoptosis Assays

#### 3.4.1. Apoptosis Analysis Using Annexin V and PI Staining

The Annexin V-FITC detection kit I (BD Biosciences, Franklin Lakes, NJ, USA) was applied to assess the amount of apoptotic and necrotic cells in compound **4**-treated and untreated MCF-7 cells over 24 h of treatment, as previously stated [36,37,38]. In brief, MCF-7 cells were collected, plated in 6-well culture dishes with 1 × 10^6^ cells per well, and cell adhesion and proliferation were then observed overnight.

The MCF-7 cells were subsequently subjected to compound **4** for a short period of time (24 h). Trypsinization, centrifugation, a PBS wash, and the addition of the Annexin V binding buffer were followed by the addition of Annexin V-FITC and propidium iodide dyes to both compound **4**-treated and untreated MCF-7 cells. Finally, a flow cytometer (BD Biosciences) was employed to visualize the outcomes by the number of apoptotic cells across the different stages.

#### 3.4.2. DNA Fragmentation Analysis Using a Comet Assay

Prior to subjecting the MCF-7 cells to compound **4**, they were exposed to a caspase-3 inhibitor (Z-DEVD-FMK) in order to assess the potential involvement of caspase-3 activity in the DNA damage induced by compound **4**. The occurrence of DNA damage was assessed in MCF-7 cells treated with compound **4** only (IC_50_ concentration) and MCF-7 cells that were pretreated with Z-DEVD-FMK (50 μM) for 1 h and then treated with compound **4** (IC_50_ concentration) for 24 h, utilizing the comet test [39,40]. Fragmentation causes the broken DNA to be separated from the healthy cellular DNA, generating a comet tail that can be identified under a fluorescence microscope. Comet parameters were calculated using TriTek Comet Score™ software (TriTek Corp., Herndon, VA, USA), following a computerized image analysis system that recorded 100 photographs of randomly shaped comets for each slide. The tail moment and tail DNA, two often-employed markers, were combined for data finding. The Olive tail moment (OTM) has been honed to be the most precise method for assessing DNA damage. In accordance with the following Equation (2), its value can be determined by the tail’s DNA concentration and DNA mobility [30].

OTM = quantity of tail moment × quantity of tail DNA/100(2)

### 3.5. Apoptosis: Underlying Mechanisms

#### 3.5.1. Analysis of the Cell Cycle’s Distinct Stages

Cell cycle testing, which makes use of the DNA content of the tested cells, was used to distinguish between the various stages of cell division. As described before [40,41], a flow cytometry kit (Abcam, Cambridge, UK, catalog ab139418) was applied to compare the percentage of untreated and treated MCF-7 cells with an IC_50_ concentration of compound **4** across the cell cycle. In each well of a 6-well plate, 1 × 10^6^ MCF-7 cells were located and incubated overnight. Subsequently, after 24 h, these prepared cells were subjected to compound **4**.

Following collection, trypsinization, and overnight incubation in cold ethanol, the cells were examined. Following a 30 min incubation period at 37 degrees Celsius in the dark with propidium iodide mixture, the cells were stained. In the end, DNA measurement with flow cytometry (BD FACSCalibur™) was used to record the percentage of cells in each cell cycle phase. A histogram was used to depict the data that were gathered.

#### 3.5.2. ELISA Examination for Quantifying the Caspase-3 Levels

The expression levels of the human active caspase-3 protein when cleaved at Asp175/Ser176 were measured in compound **4**-treated MCF-7 cells, using an ELISA method (Invitrogen, Waltham, MA, USA, Catalog # KHO1091). In a nutshell, after incubating the tested compound **4** with MCF-7 cell cultures for 2 h, the supernatant (100 μL) was plated into a 96-well ELISA plate, and the resulting pellets were discarded. Each well was then suspended with 100 μL of a caspase-3 (active) antibody detection solution and left to incubate at room temperature for 1 h. The solution was then decanted from the wells and rinsed four times. Furthermore, HRP-Anti-Rabbit Antibody solution (100 µL) was added to each well, and the plate was incubated for 30 min at room temperature. A stabilized chromogen substrate (100 µL) was transferred to each well, and the plate was allowed to incubate for 30 min at room temperature, in darkness. At 450 nm, optical density readings were taken from the tested plate after adding 100 µL of stop solution to each well. Utilizing a standard curve, the quantity of active caspase-3 protein in the treated MCF-7 cells was calculated.

## 4. Conclusions

Six new derivatives of N,N′-diarylurea and N,N′-diarylthiourea were prepared, and their molecular structures were fully characterized via spectroscopic techniques. When compared to the other compounds, the compound diarylthiourea 4 had the most potent IC_50_ concentration for the growth of human MCF-7 cancer cells. Additionally, using in vitro studies, it has been confirmed that utilizing the compound diarylthiourea 4 is safe for living tissues. The underlying mechanisms of the investigated Diarylthiourea 4 compound’s impact on the inhibiting growth of human MCF-7 cancer cells were investigated using apoptosis detection approaches (Annexin V/PI staining and comet assays), flow cytometric investigations, and the ELISA technique.

The findings suggested that diarylthiourea 4 inhibited the growth of MCF-7 cancer cells in a cytotoxic manner and that this effect may have been mediated by S-phase cell cycle arrest and caspase-3 activation via an intrinsic apoptotic route. These findings imply that the Diarylthiourea 4 chemical could potentially be employed to treat breast cancer, even if more research utilizing in vivo animal experimental models is necessary.

## Figures and Tables

**Table 1:** Comet analysis parameters were applied for assessing the variations in DNA damage between compound **4**-treated MCF-7 cells with and without caspase-3 inhibitor pretreatment and untreated MCF-7 cells. The findings of three separate tests are given as mean ± SD, *** *p* < 0.001 and ** *p* < 0.01.

The Tested Cells	Tail Length (PX)	% DNA in Tail	Tail Moment	Olive Tail Moment (OTM)
The untreated control MCF-7 cells	7.52 ± 1.82	3.11 ± 1.73	0.22 ± 0.02	0.55 ± 0.02
The treated MCF-7 cells with compond 4 without caspase-3 inhibitor pretreatment	6.67 ± 1.94	17.26 ± 2.34 ***	1.72 ± 0.05 ***	2.38 ± 0.06 ***
The treated MCF-7 cells with compound 4 with caspase-3 inhibitor pretreatment	7.13 ± 2.21	14.37 ± 1.33 ***	1.04 ± 0.04 ***	1.87 ± 0.34 **

## Data Availability

Not applicable.

## Supplementary Materials

The following supporting information can be downloaded at: https://www.mdpi.com/article/10.3390/molecules28176420/s1, IR, ^1^H, ^13^C NMR spectra, and mass spectrometry of compounds **1**–**6**.

## Sample Availability

Samples of the compounds **1**–**6** are available from authors mohamed.elatawi@alexu.edu.eg (M.A.E.-A.) and ahoda@sci.cu.edu.eg (H.A.A.)
