# Supplementary material for: Synthesis, Characterization, and Anticancer Activity of New N,N′-Diarylthiourea Derivative against Breast Cancer Cells
Source: Molecules. 2023 Sep 3;28(17):6420. doi: 10.3390/molecules28176420 (PMC10490226; doi:10.3390/molecules28176420)
Supplement: Supplementary file 1 [file molecules-28-06420-s001.zip › molecules-2542585-supplementary.pdf]

Synthesis, Characterization, and Anticancer Activity of New N,N′-Diarylthiourea Derivative against Breast Cancer Cells

Mohamed A. El-Atawy [1,2], Mai S. Alsubaie [1], Mohammed L. Alazmi [1], Ezzat A. Hamed [1,*], Demiana H. Hanna [3,*], Hoda A. Ahmed [3] and Alaa Z. Omar [1]

1 Chemistry Department, Faculty of Science, Alexandria University, P.O. Box 426 Ibrahemia, Alexandria 21321, Egypt; mohamed.elatawi@alexu.edu.eg (M.A.E.-A.); maisaudalsubaie@gmail.com (M.S.A.); mohammadalazmmi@gmail.com (M.L.A.); alaazaki@alexu.edu.eg (A.Z.O.)

2 Chemistry Department, Faculty of Science, Taibah University, Yanbu 46423, Saudi Arabia

3 Department of Chemistry, Faculty of Science, Cairo University, Giza 12613, Egypt; ahoda@sci.cu.edu.eg

* Correspondence: ezzat.awad@alexu.edu.eg (E.A.H.); dhelmy@sci.cu.edu.eg (D.H.H.)

Table S1: Effect of the tested compounds (1- 6) on MCF-7 cell viability through the different doses (50 to 1000 µM) for 24 h incubation.

Dose (µM)	Cell Viability %					
	**Compound 1**	**Compound 2**	**Compound 3**	**Compound 4**	**Compound 5**	**Compound 6**
**1000**	36.41± 1.34	23.32± 2.33	20.93± 2.75	7.28± 2.56	17.98± 1.45	36.73± 1.66
**600**	57.24± 2.44	38.27± 1.97	56.73± 2.55	18.31± 1.92	45.37± 2.74	46.91± 2.34
**300**	67.17± 1.13	49.86± 1.77	64.92± 1.66	21.21± 1.66	61.98± 1.84	79.23± 2.64
**150**	80.62± 2.76	72.24± 2.13	80.68± 2.44	48.14± 1.15	79.45± 1.92	84.11± 1.83
**100**	87.32± 1.34	88.57± 2.22	93.59± 1.82	90.23± 2.78	96.45± 2.33	91.71± 2.47
**50**	98.58± 2.21	98.76± 2.26	97.28± 2.45	99.95± 2.24	98.64± 2.53	96.44± 2.63

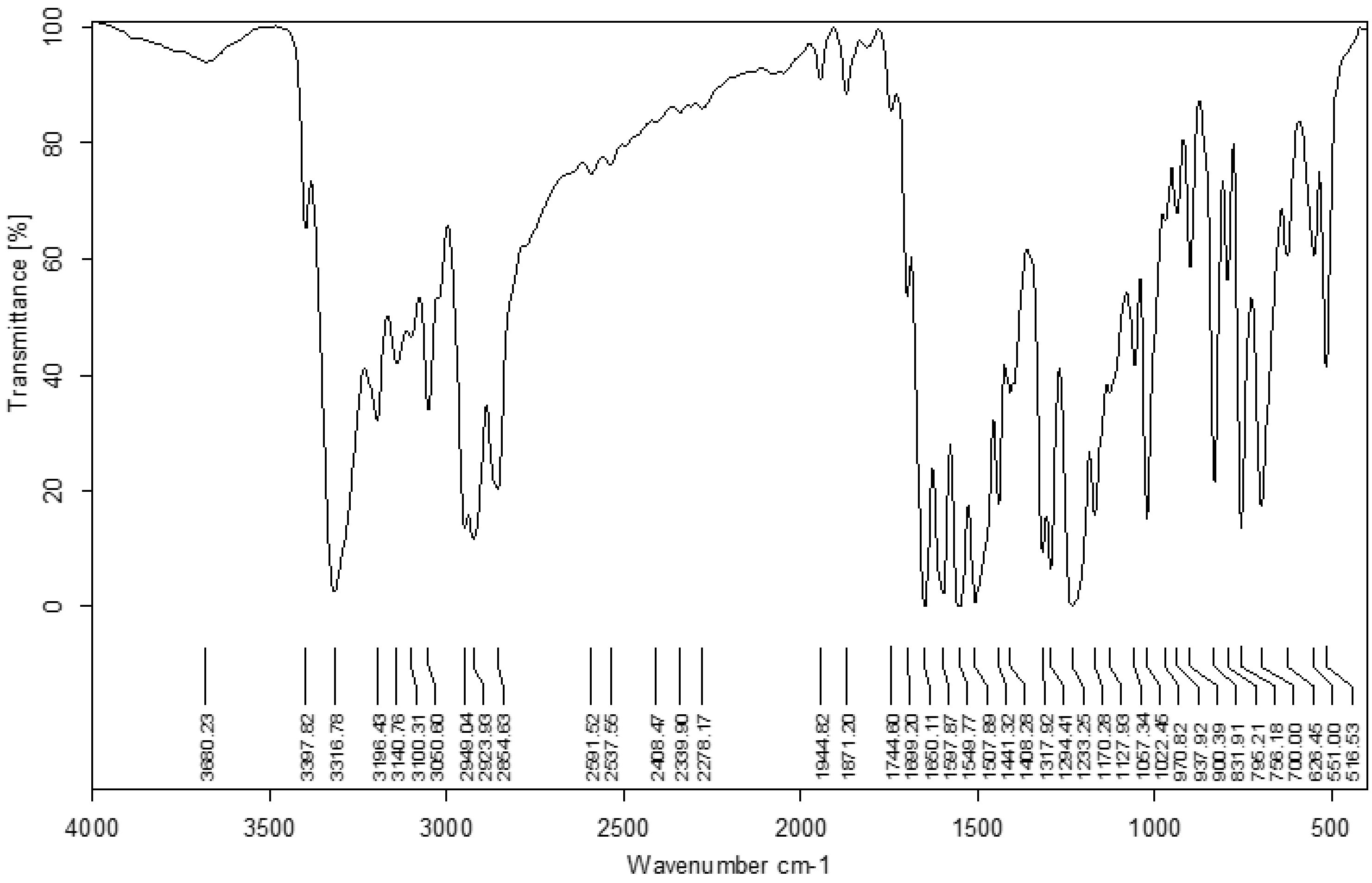

IR (KBr) spectrum of 1-(4-(hexyloxy)phenyl)-3-phenylurea **1**

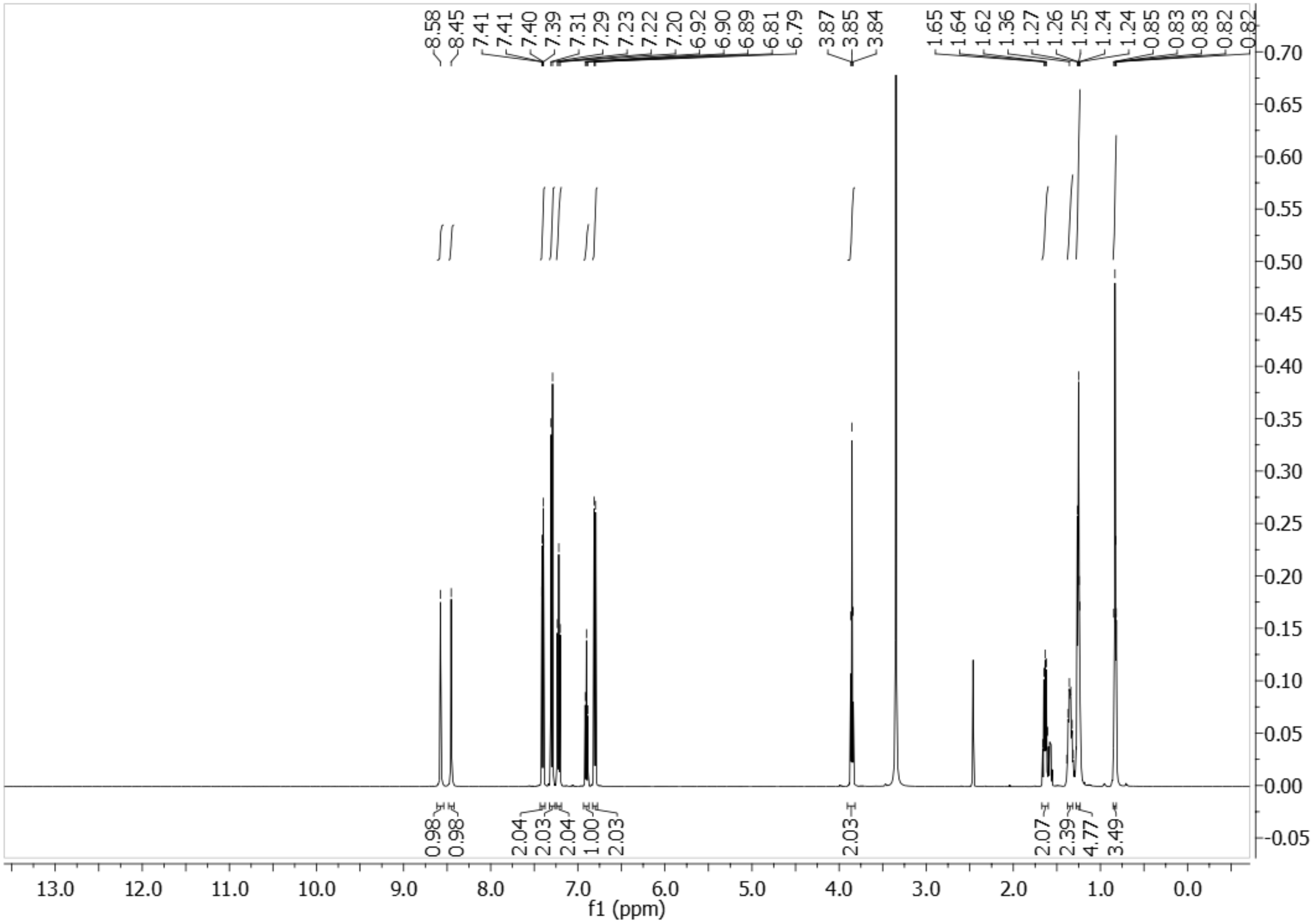

^{1}H NMR (DMSO-*d6*) spectrum of 1-(4-(hexyloxy)phenyl)-3-phenylurea **1**

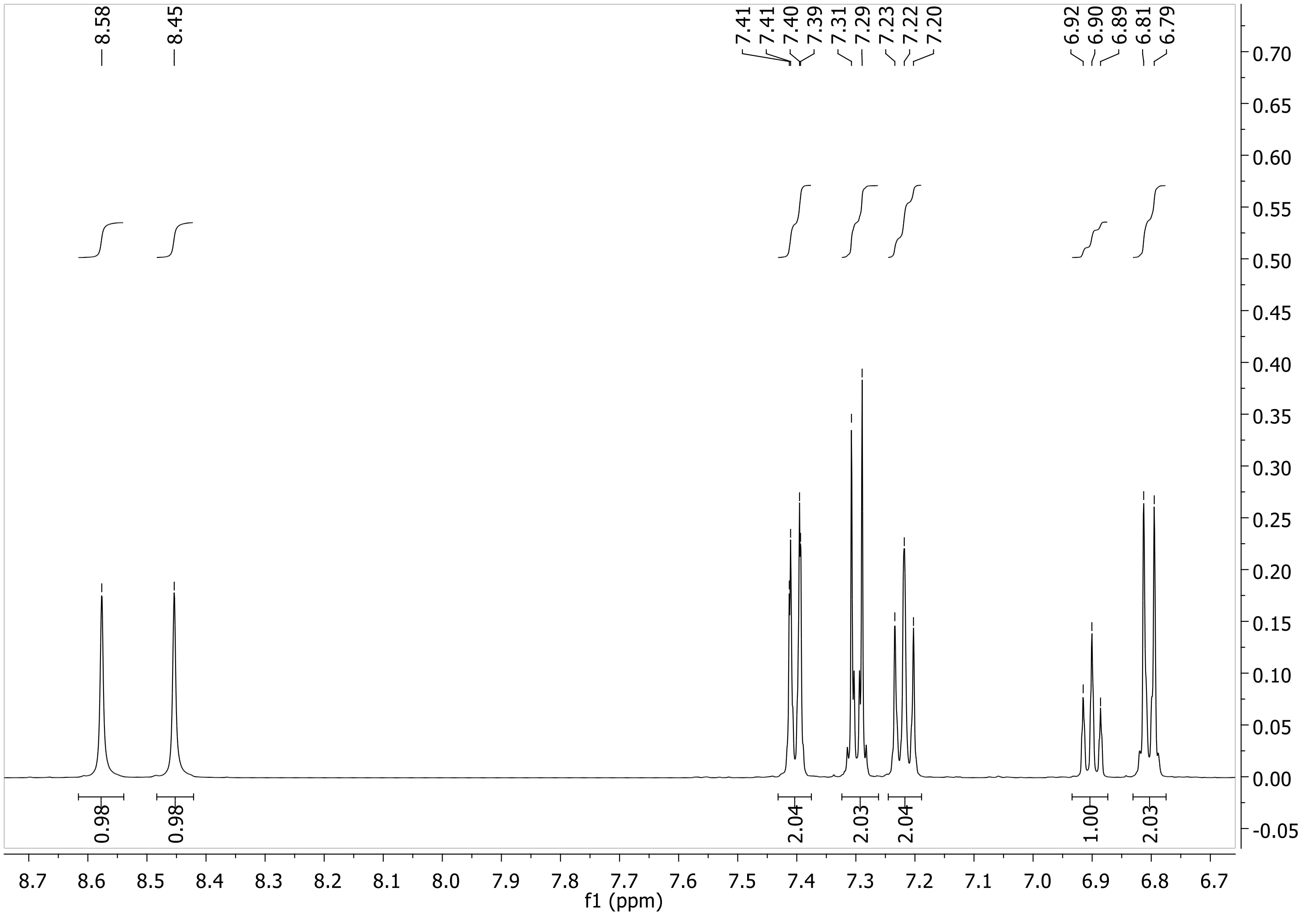

^{1}H NMR (DMSO-*d6*) spectrum of 1-(4-(hexyloxy)phenyl)-3-phenylurea **1**

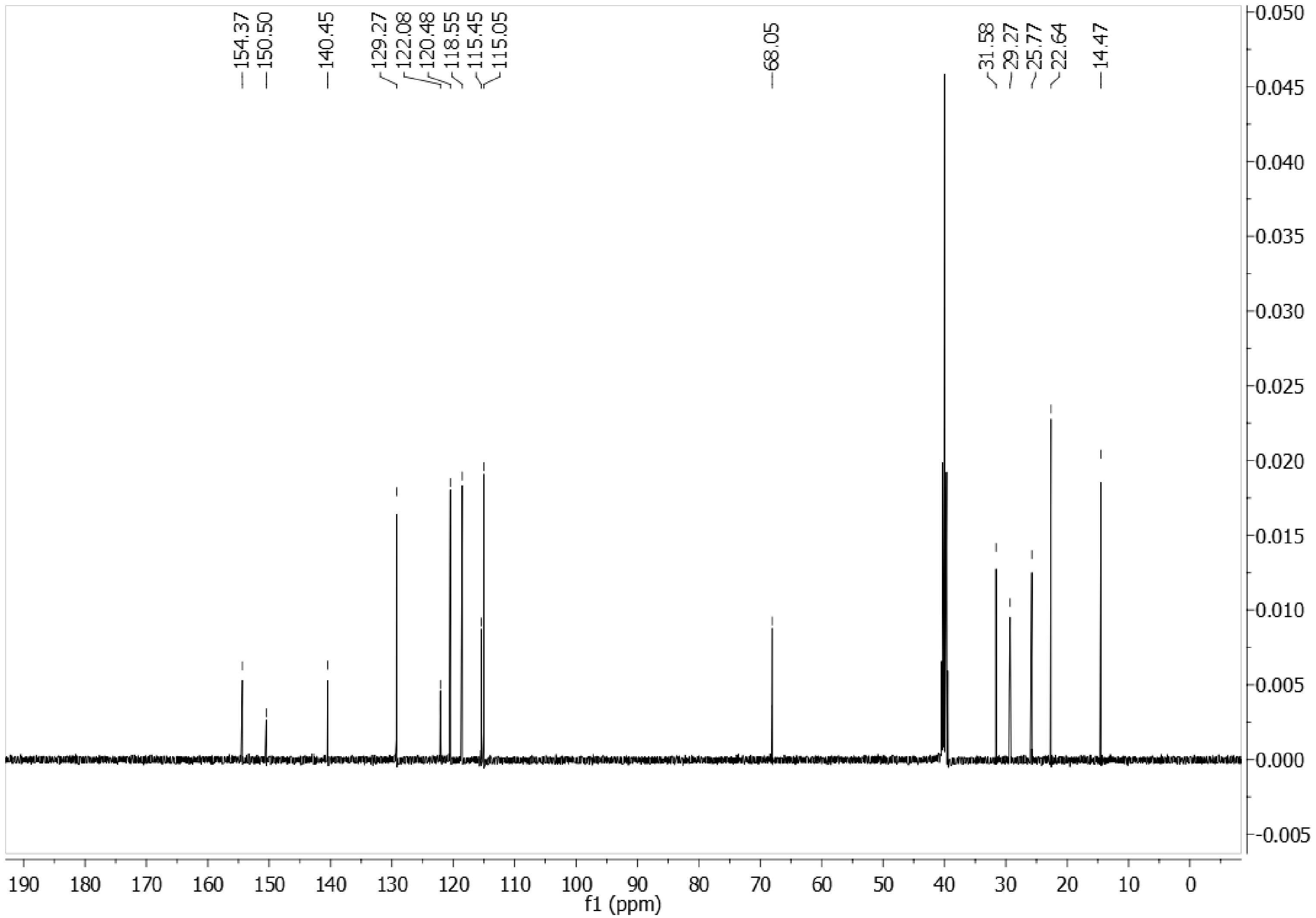

^{13}C NMR (DMSO-*d6*) of 1-(4-(hexyloxy)phenyl)-3-phenylurea **1**

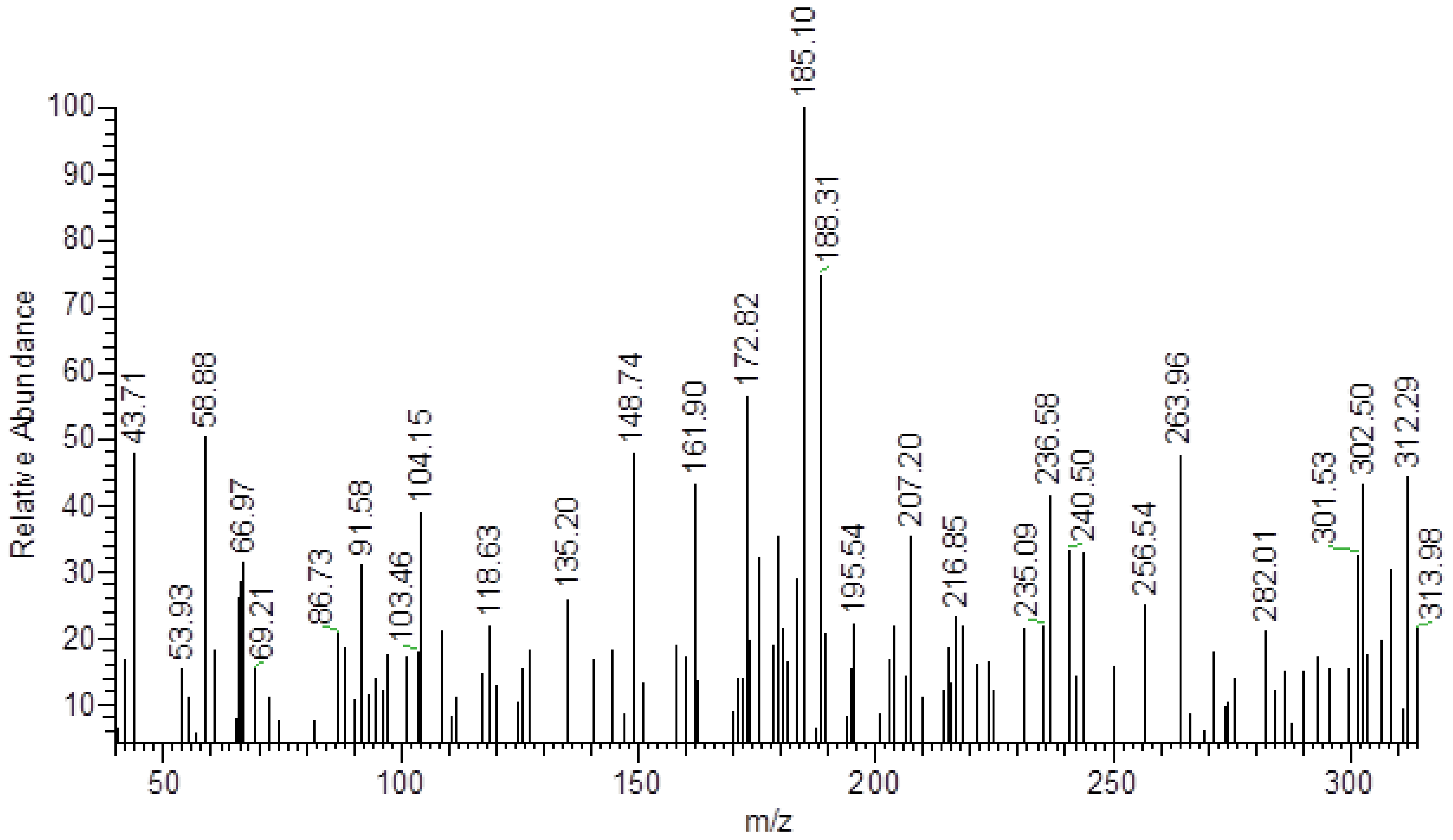

Mass spectrum of 1-(4-(hexyloxy)phenyl)-3-phenylurea **1**

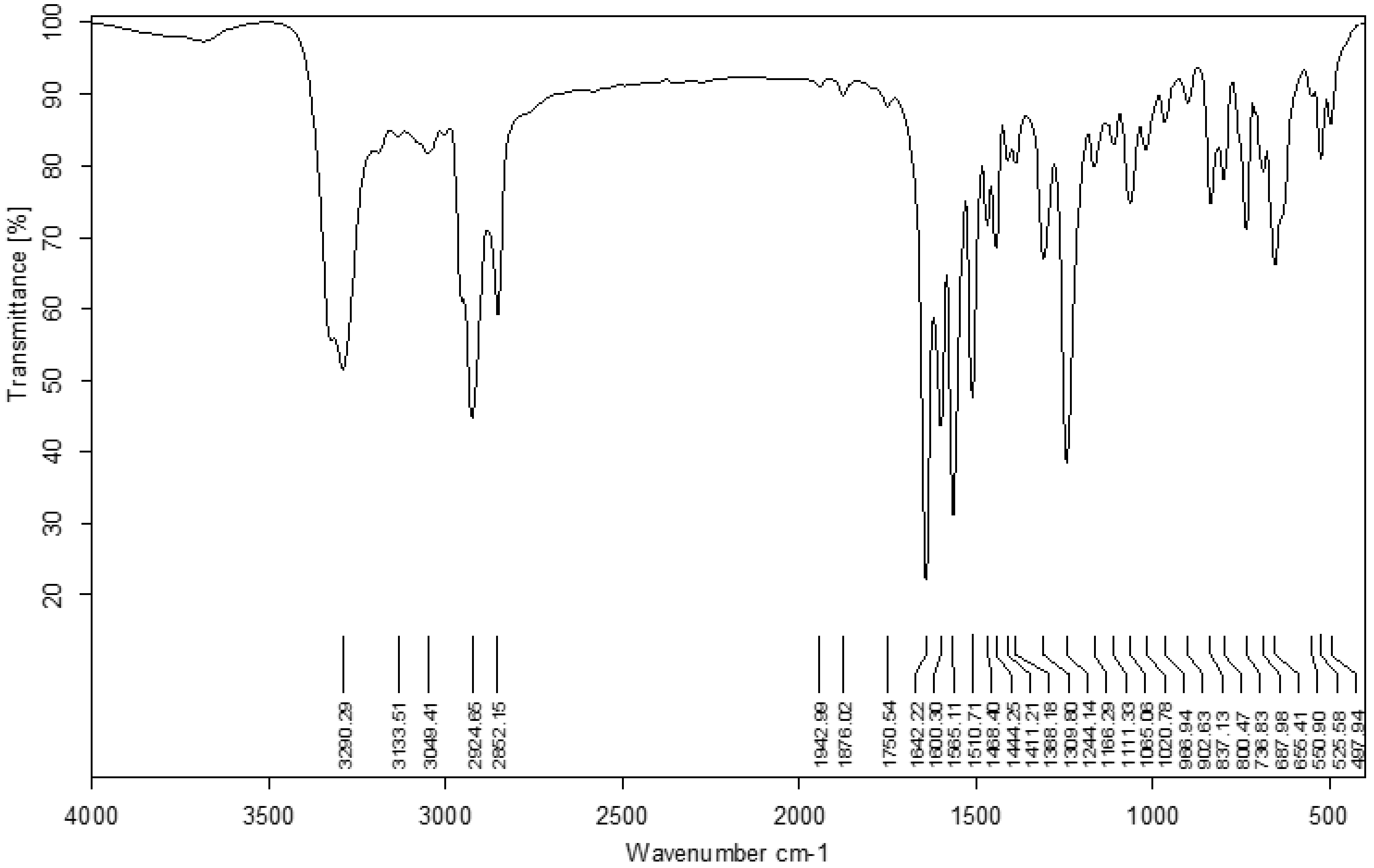

IR (KBr) spectrum of 1-(4-(octyloxy)phenyl)-3-phenylurea **2**

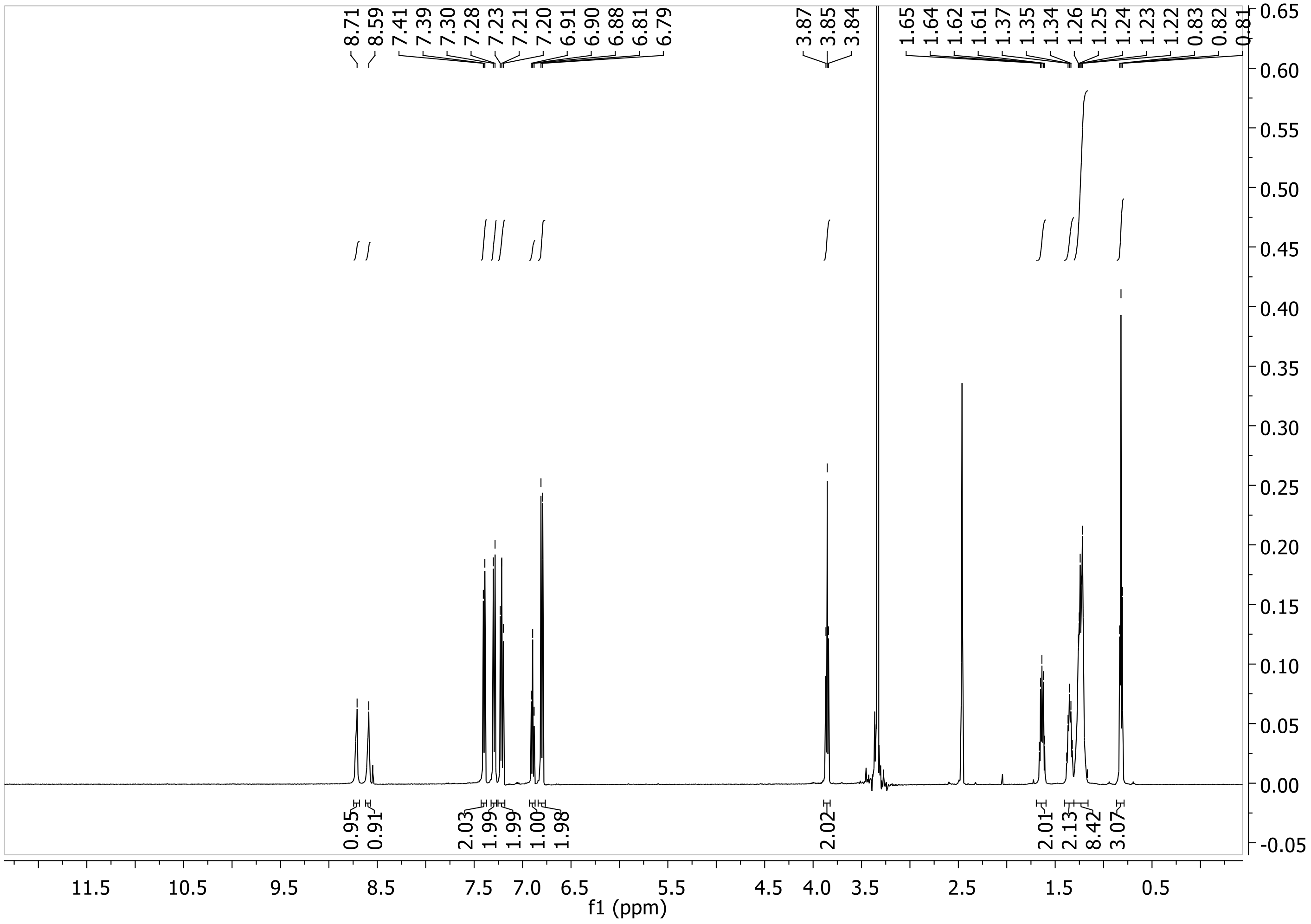

^{1}H NMR (DMSO-*d6*) spectrum of 1-(4-(octyloxy)phenyl)-3-phenylurea **2**

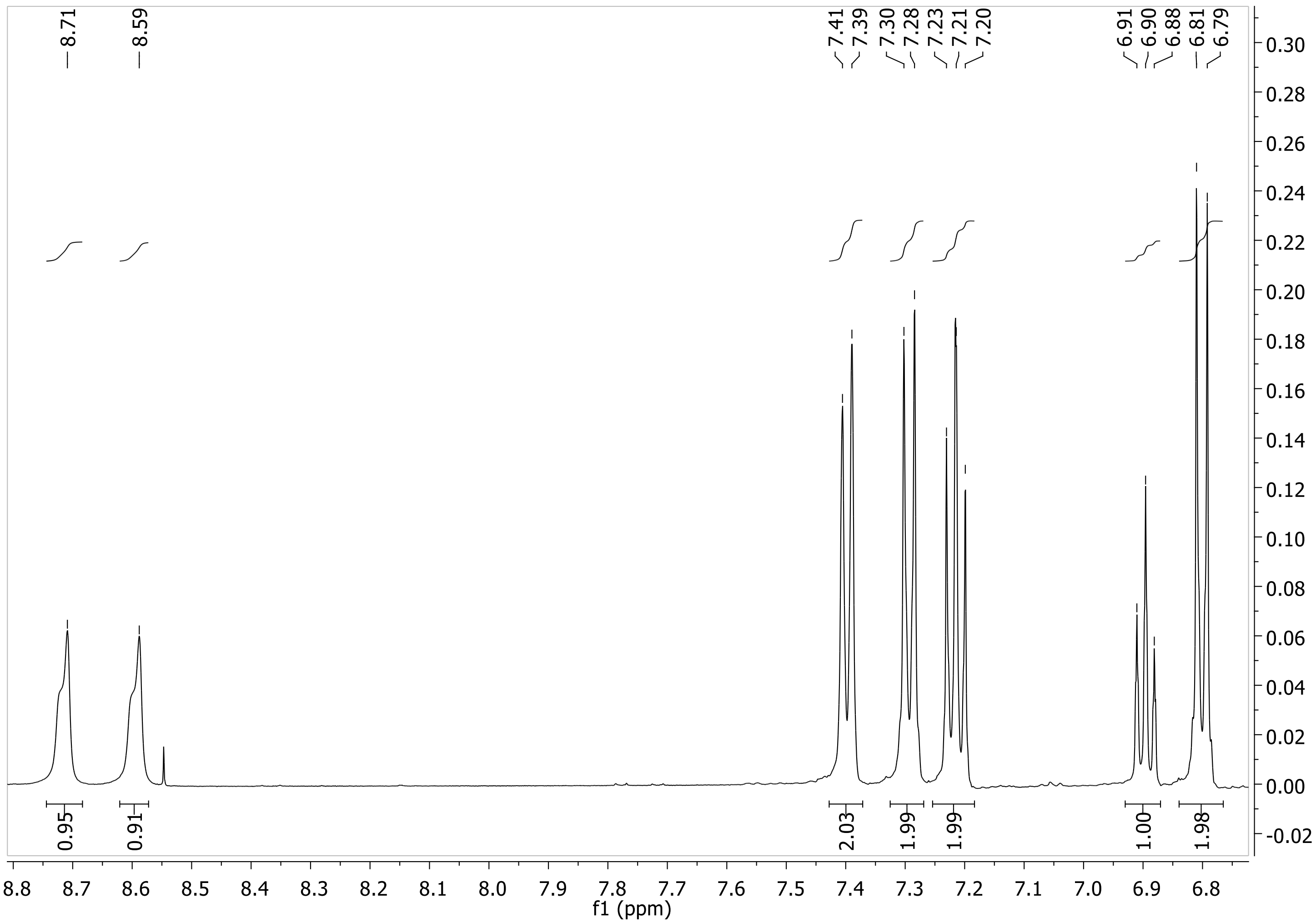

^{1}H NMR (DMSO-*d6*) spectrum of 1-(4-(octyloxy)phenyl)-3-phenylurea **2**

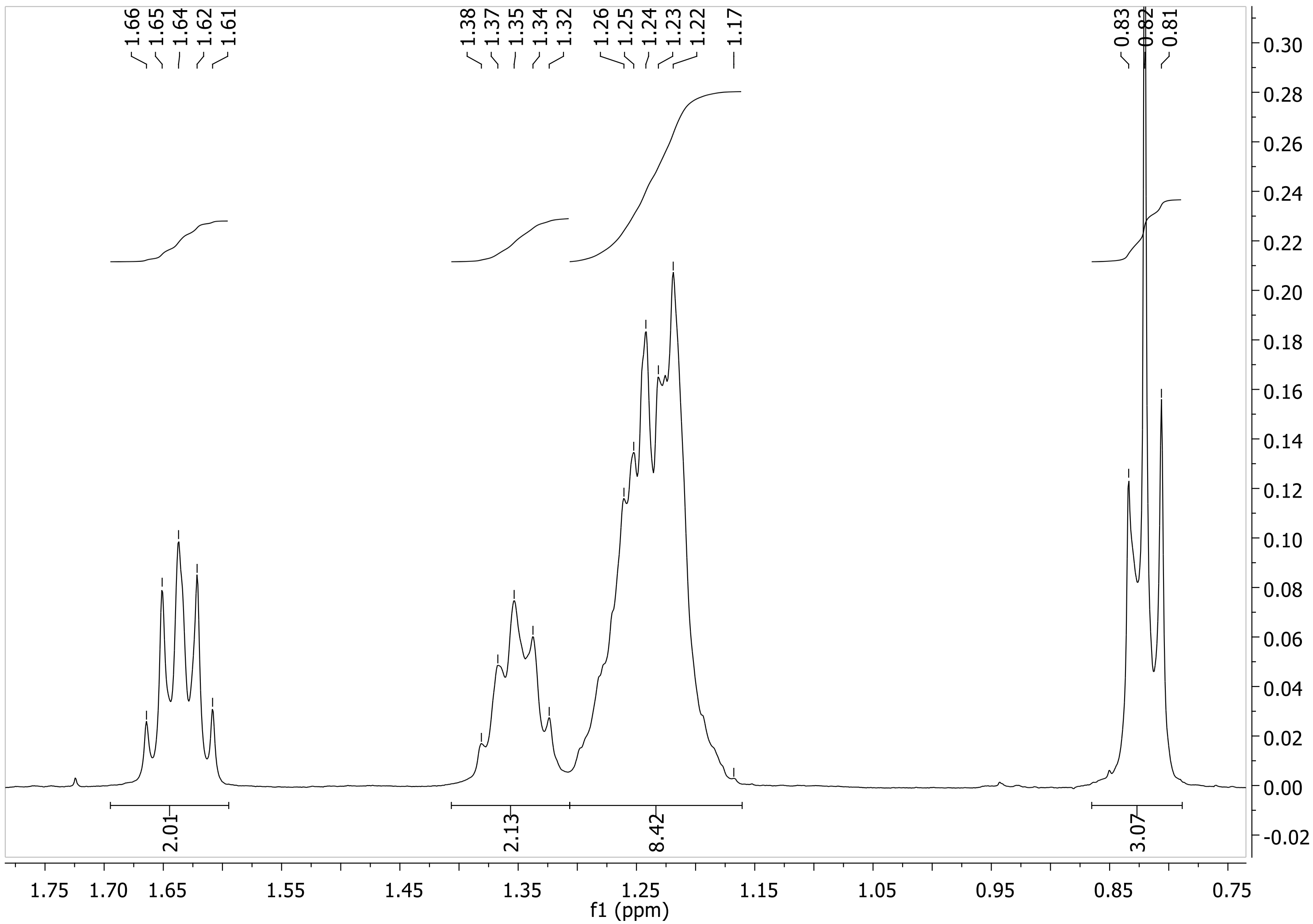

^{1}H NMR (DMSO-*d6*) spectrum of 1-(4-(octyloxy)phenyl)-3-phenylurea **2**

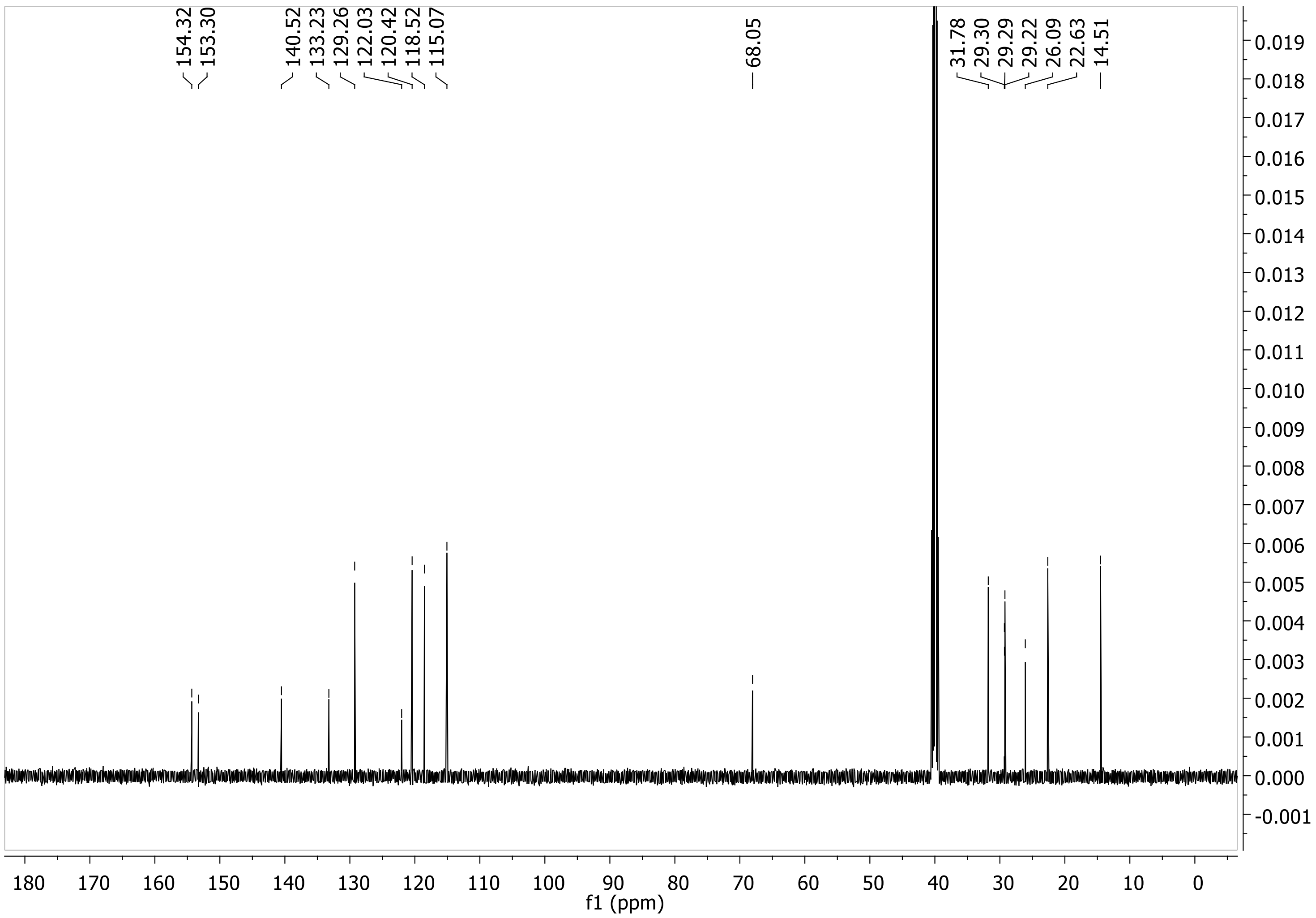

^{13}C NMR (DMSO-*d6*) of 1-(4-(octyloxy)phenyl)-3-phenylurea **2**

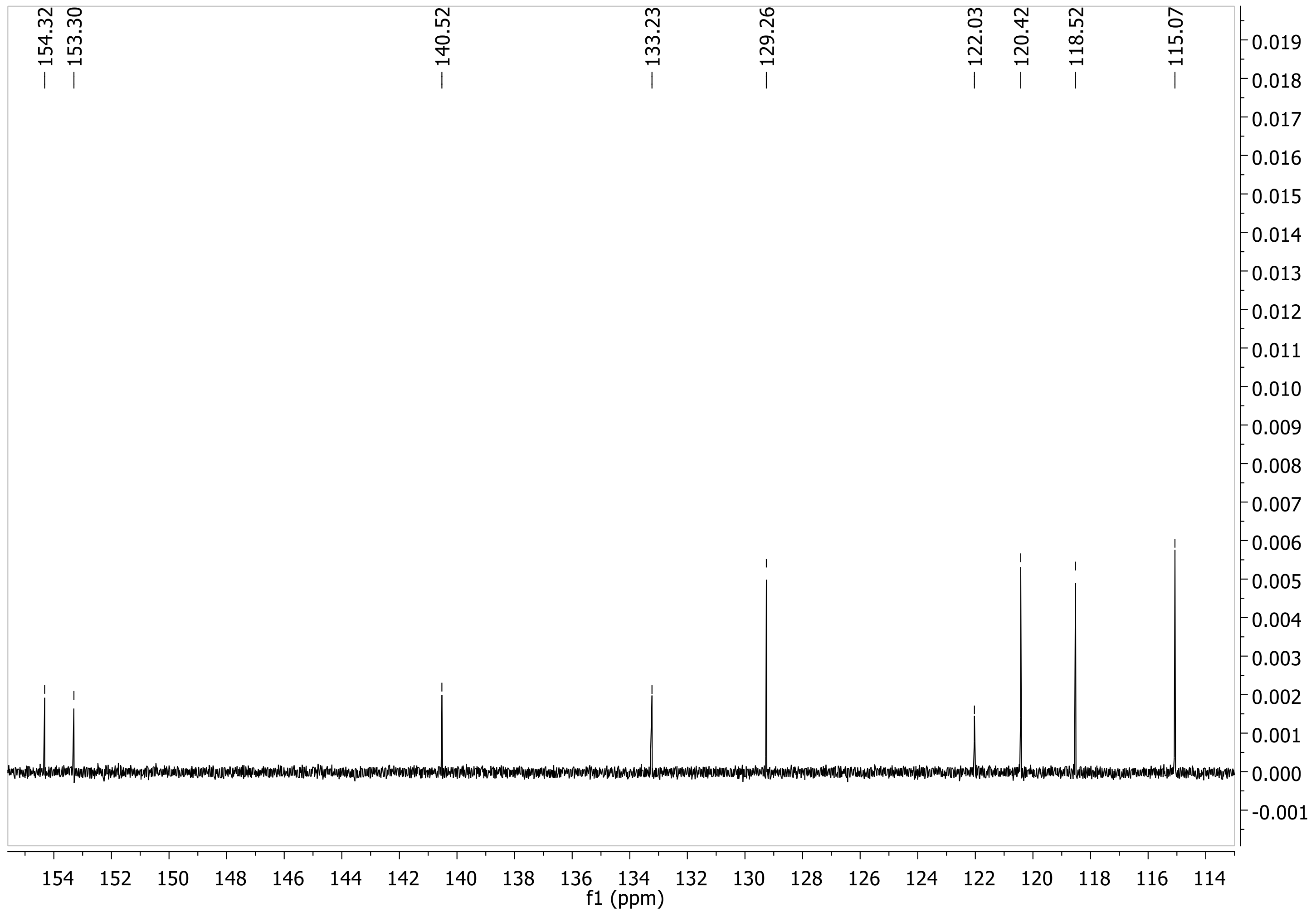

^{13}C NMR (DMSO-*d6*) of 1-(4-(octyloxy)phenyl)-3-phenylurea **2**

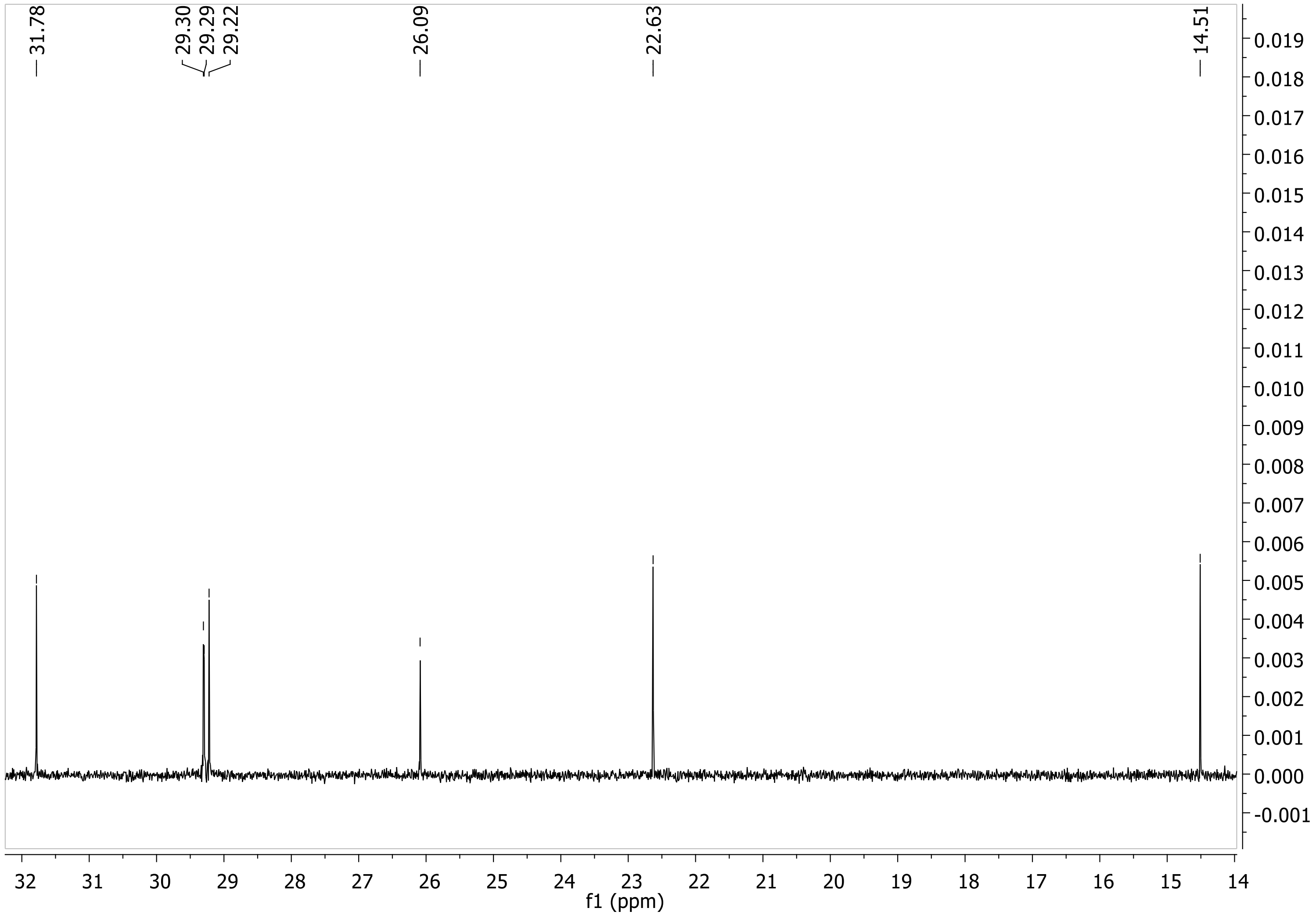

^{13}C NMR (DMSO-*d6*) of 1-(4-(octyloxy)phenyl)-3-phenylurea **2**

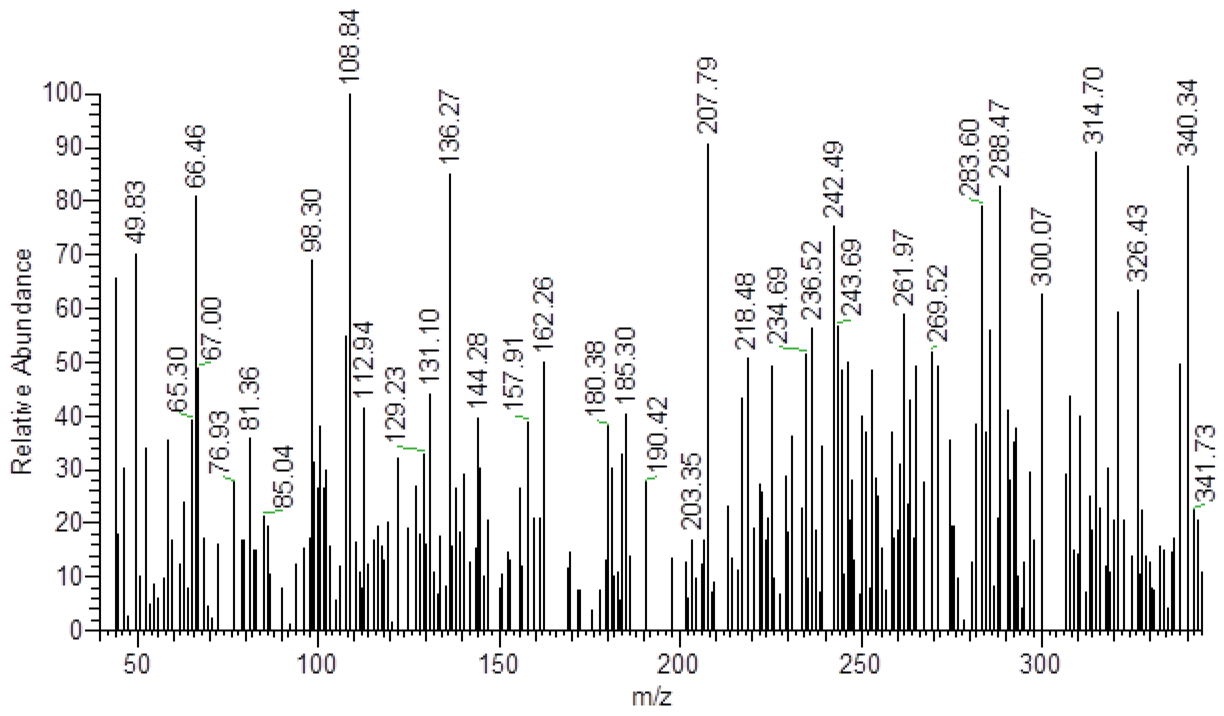

Mass spectrum of 1-(4-(octyloxy)phenyl)-3-phenylurea **2**

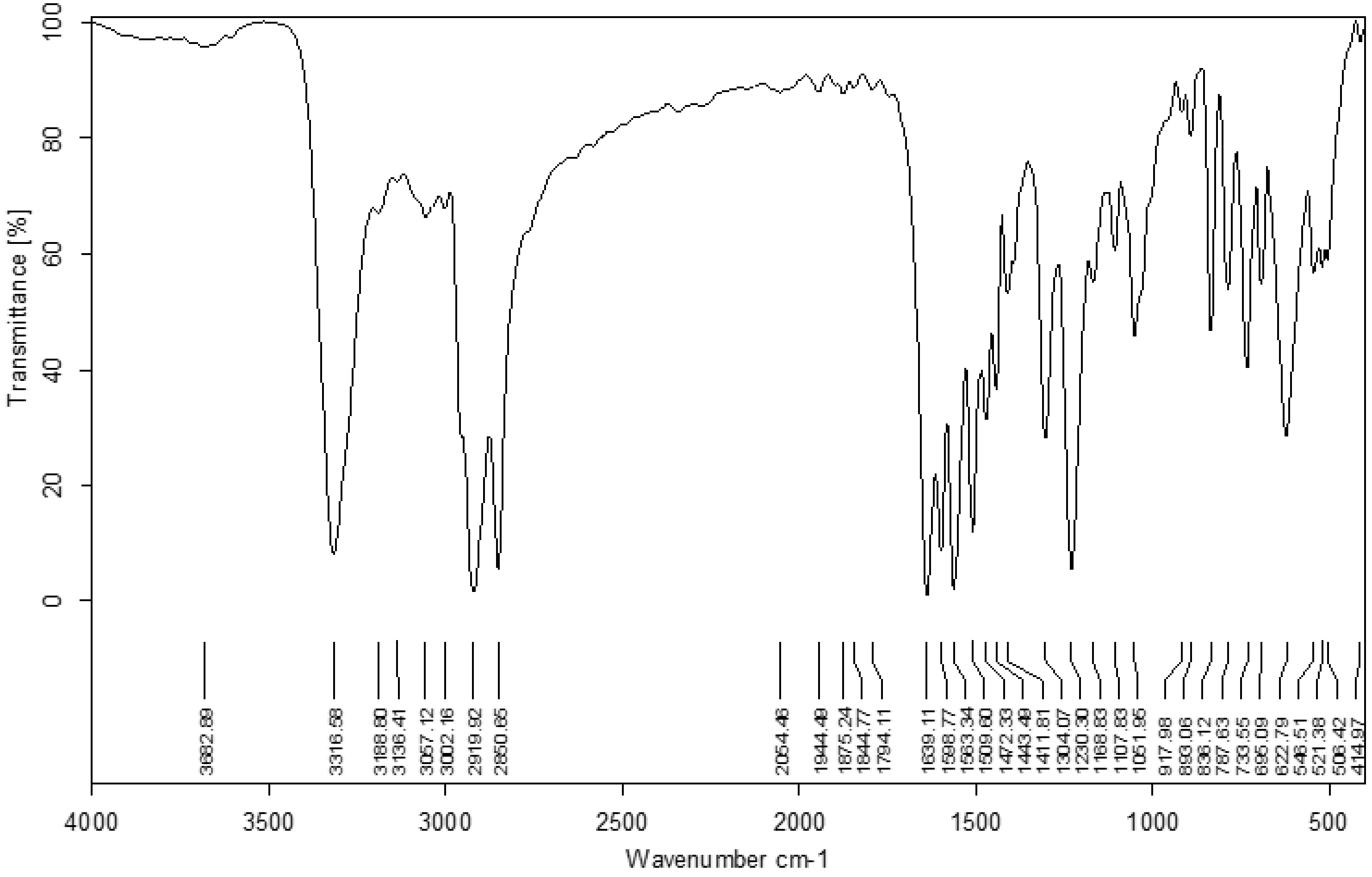

IR (KBr) spectrum of 1-(4-(hexadecyloxy)phenyl)-3-phenylurea **3**

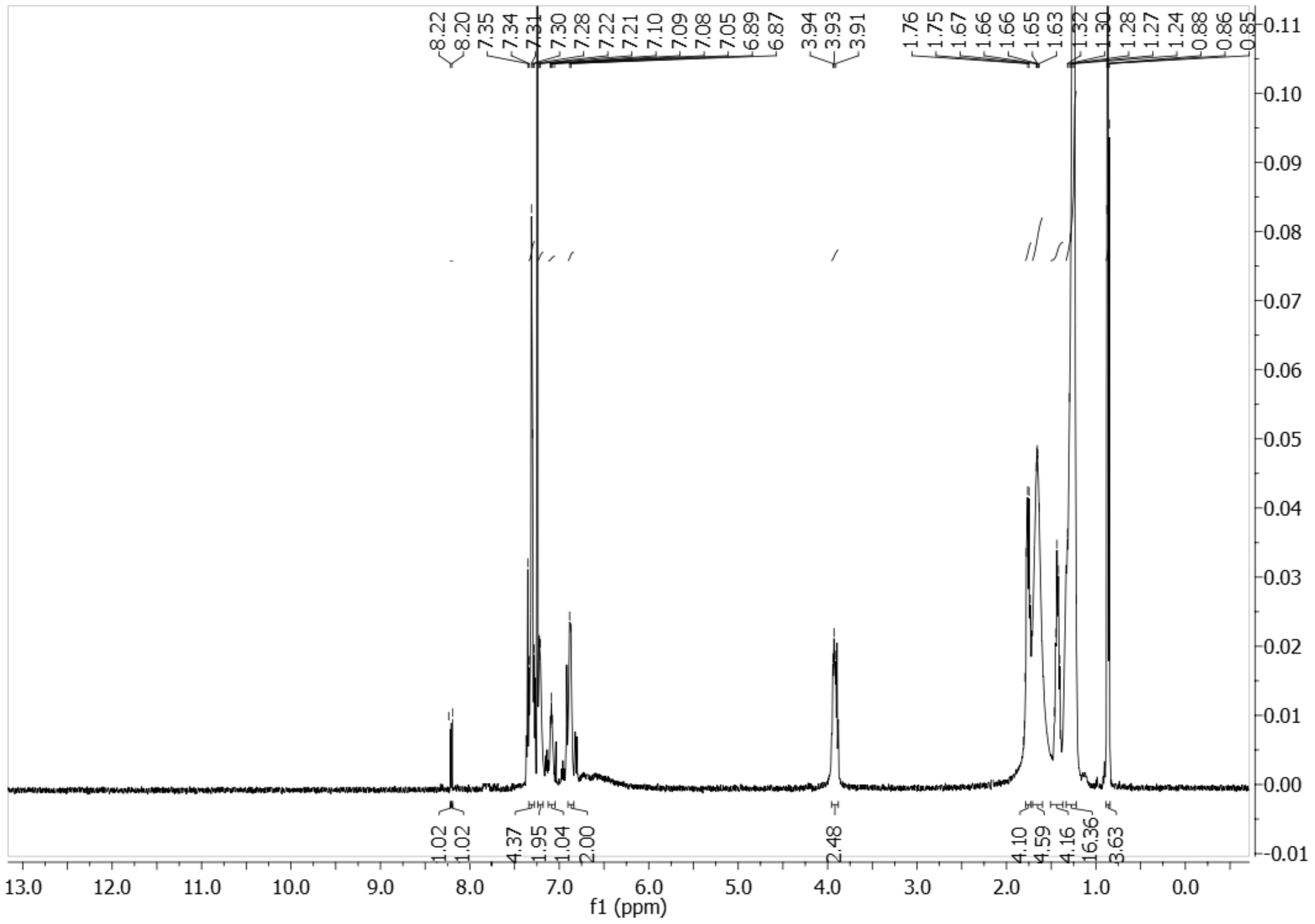

^{1}H NMR ($CDCl_3$) spectrum of 1-(4-(hexadecyloxy)phenyl)-3-phenylurea **3**

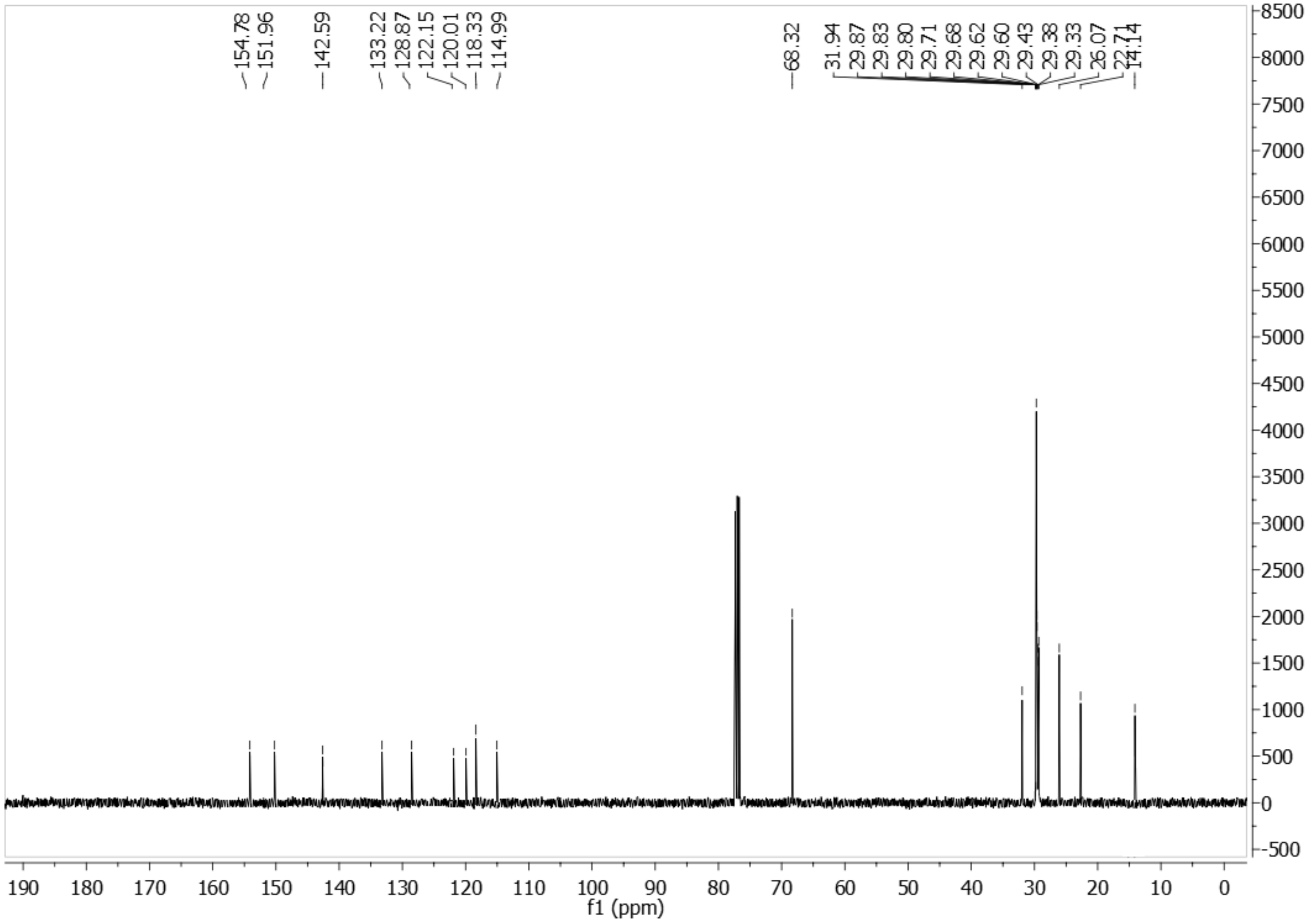

^{13}C NMR (CDCl$_3$) of 1-(4-(hexadecyloxy)phenyl)-3-phenylurea **3**

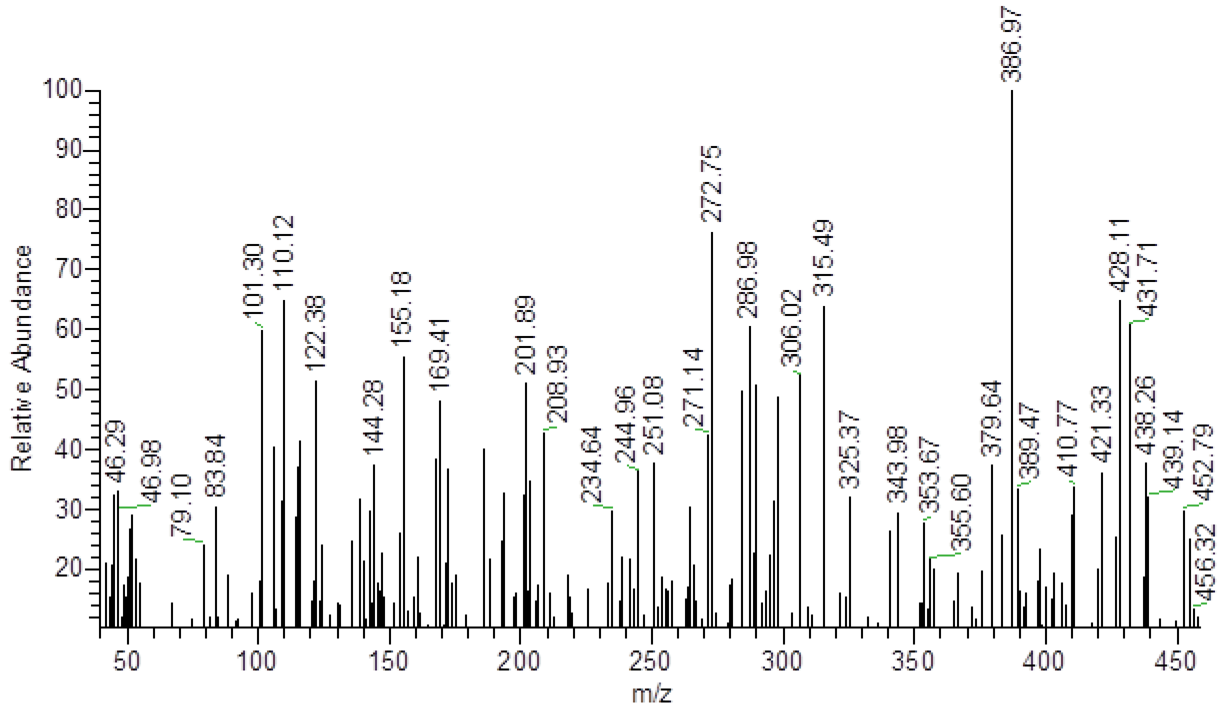

Mass spectrum of 1-(4-(hexadecyloxy)phenyl)-3-phenylurea **3**

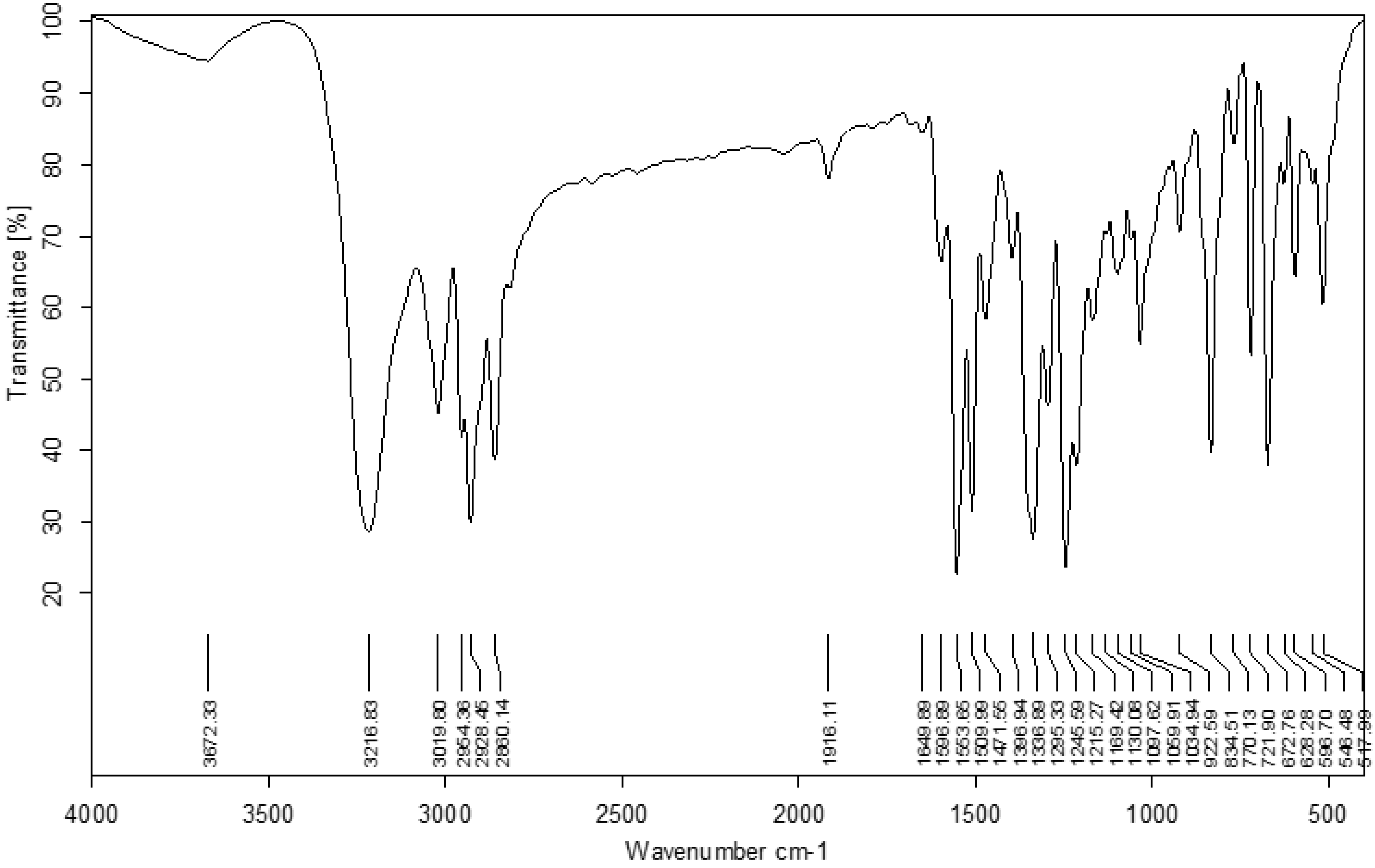

IR (KBr) spectrum of 1-(4-fluorophenyl)-3-(4-(hexyloxy)phenyl)thiourea **4**

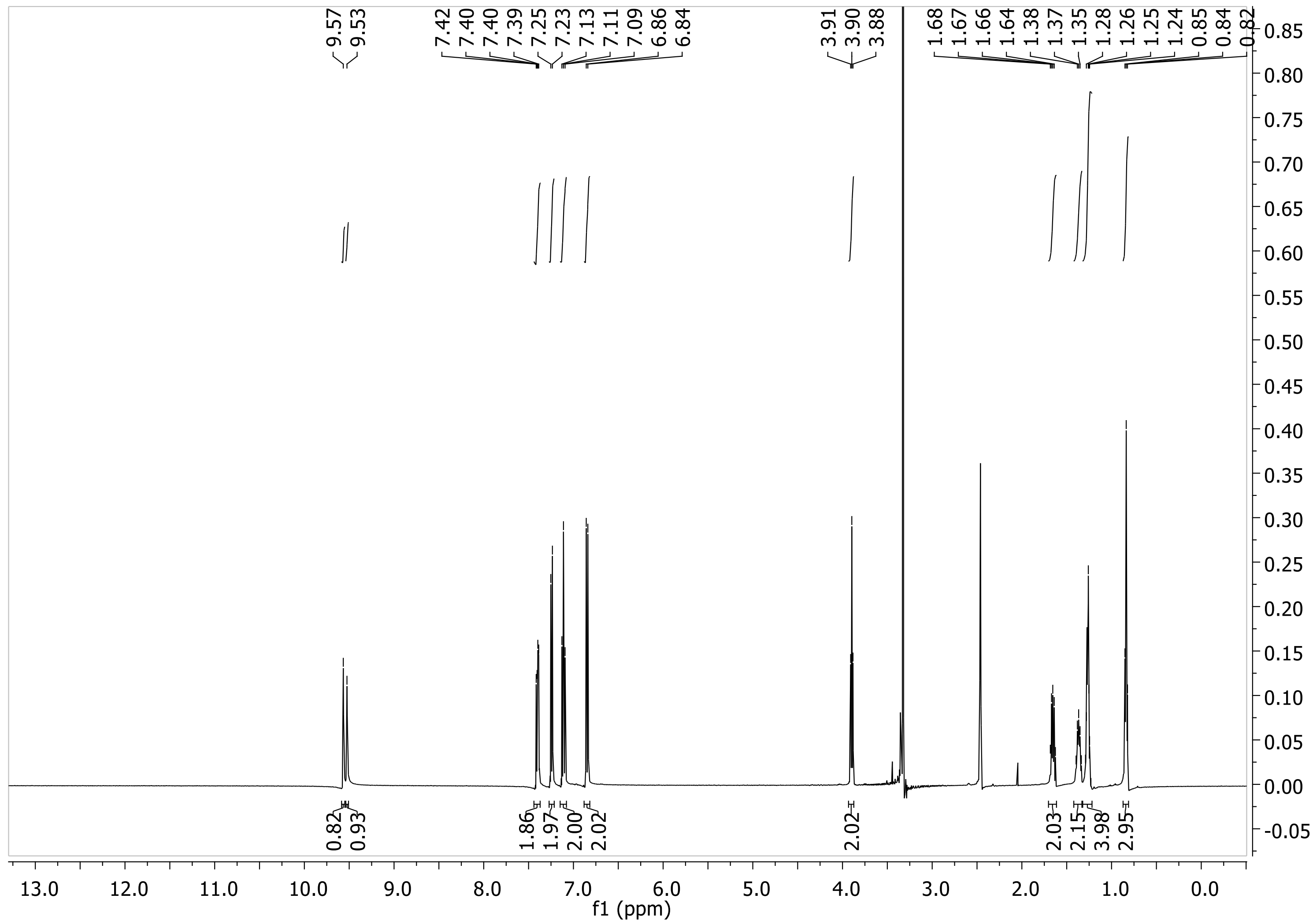

^{1}H NMR (DMSO-*d6*) spectrum of 1-(4-fluorophenyl)-3-(4-(hexyloxy)phenyl)thiourea **4**

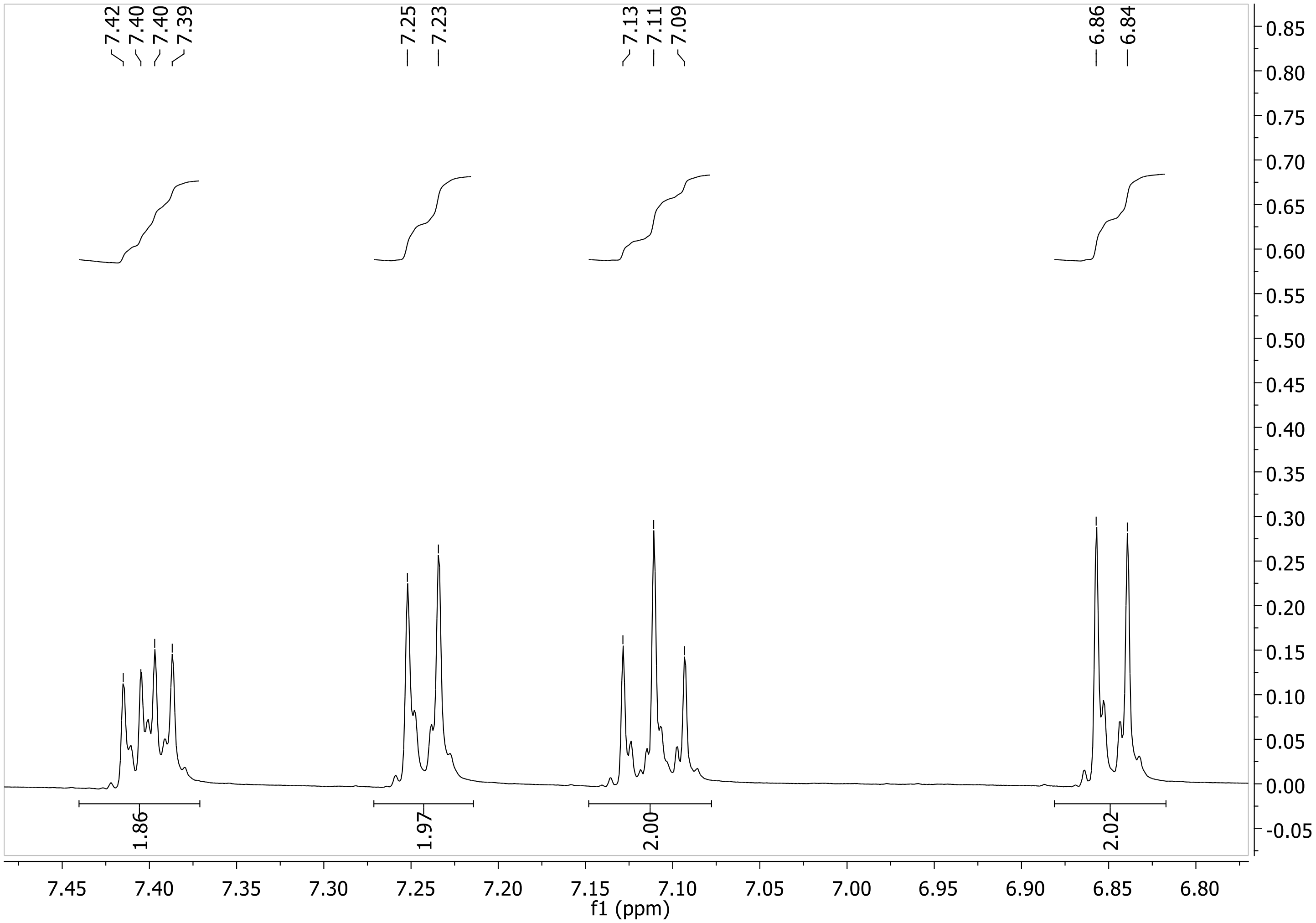

^{1}H NMR (DMSO-*d6*) spectrum of 1-(4-fluorophenyl)-3-(4-(hexyloxy)phenyl)thiourea **4**

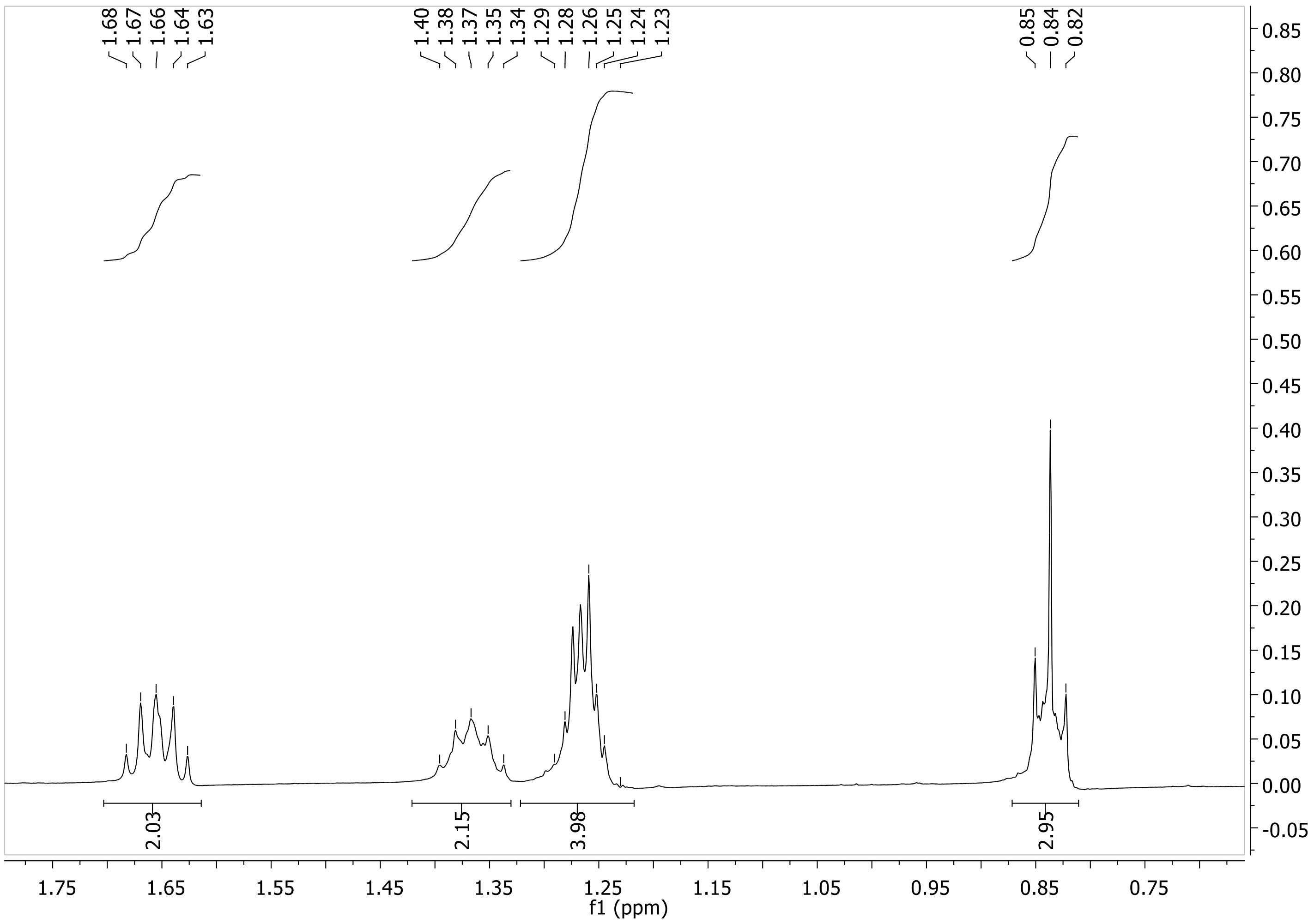

^{1}H NMR (DMSO-*d6*) spectrum of 1-(4-fluorophenyl)-3-(4-(hexyloxy)phenyl)thiourea **4**

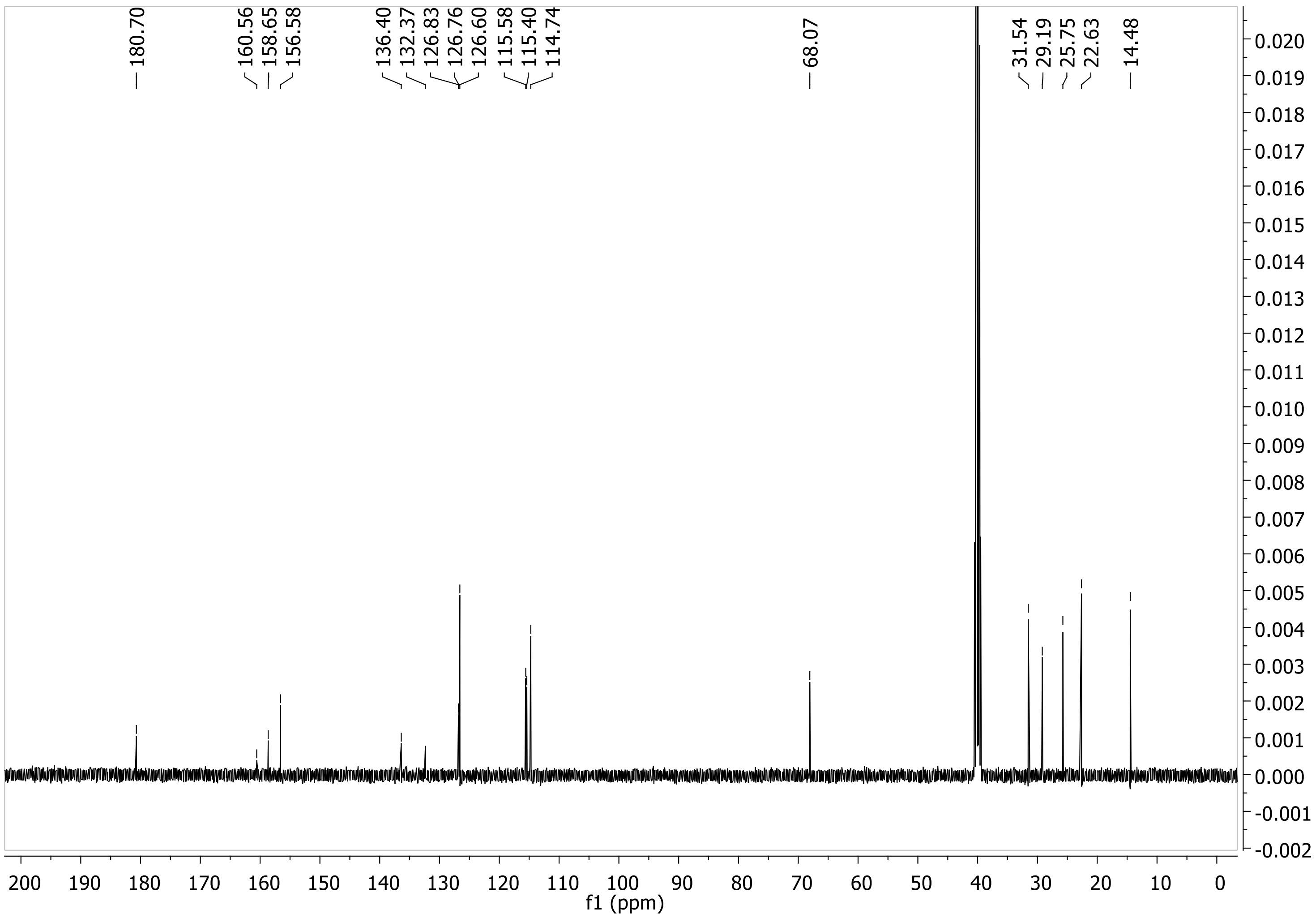

^{13}C NMR (DMSO-*d6*) of 1-(4-fluorophenyl)-3-(4-(hexyloxy)phenyl)thiourea **4**

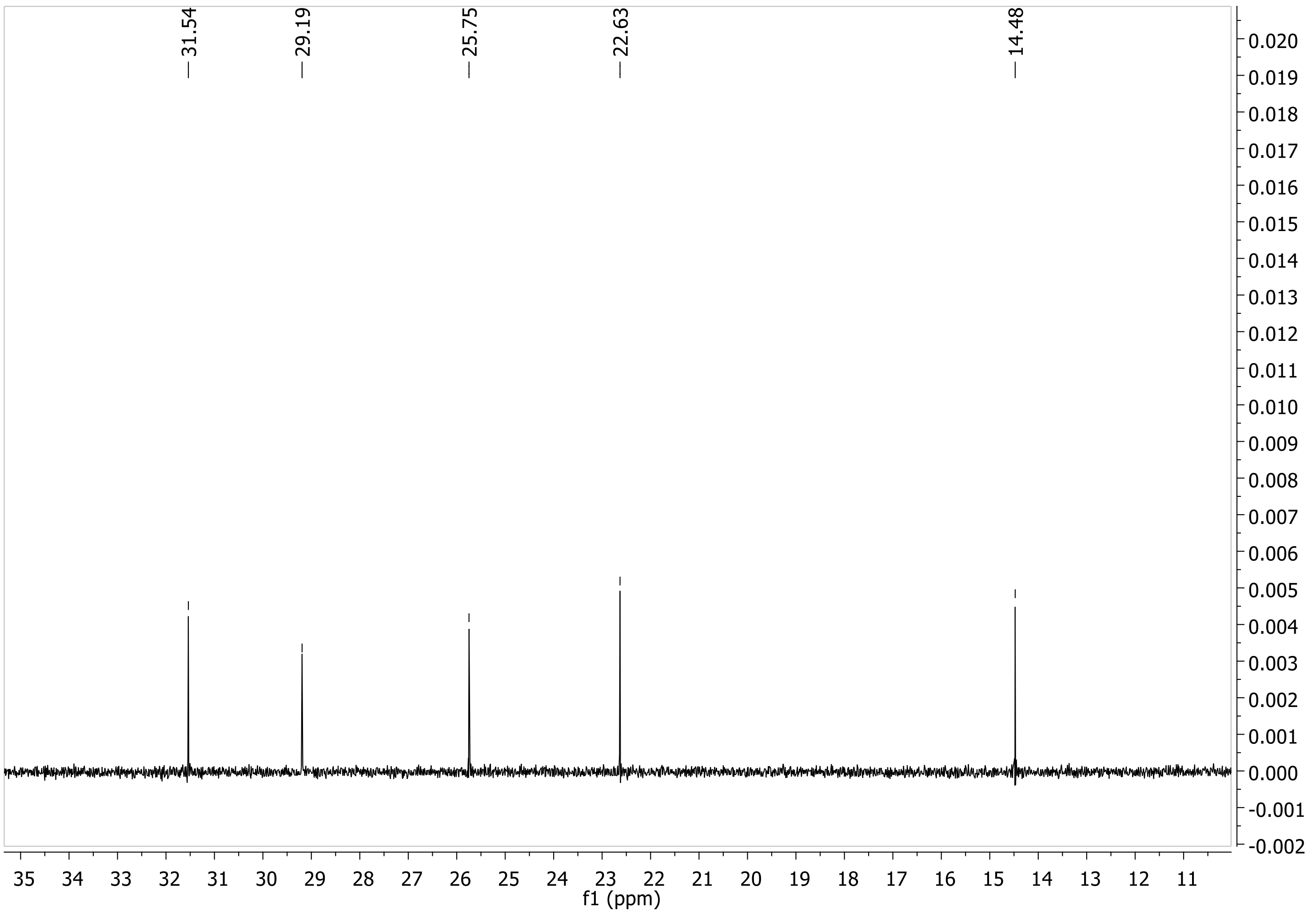

^{13}C NMR (DMSO-*d6*) of 1-(4-fluorophenyl)-3-(4-(hexyloxy)phenyl)thiourea **4**

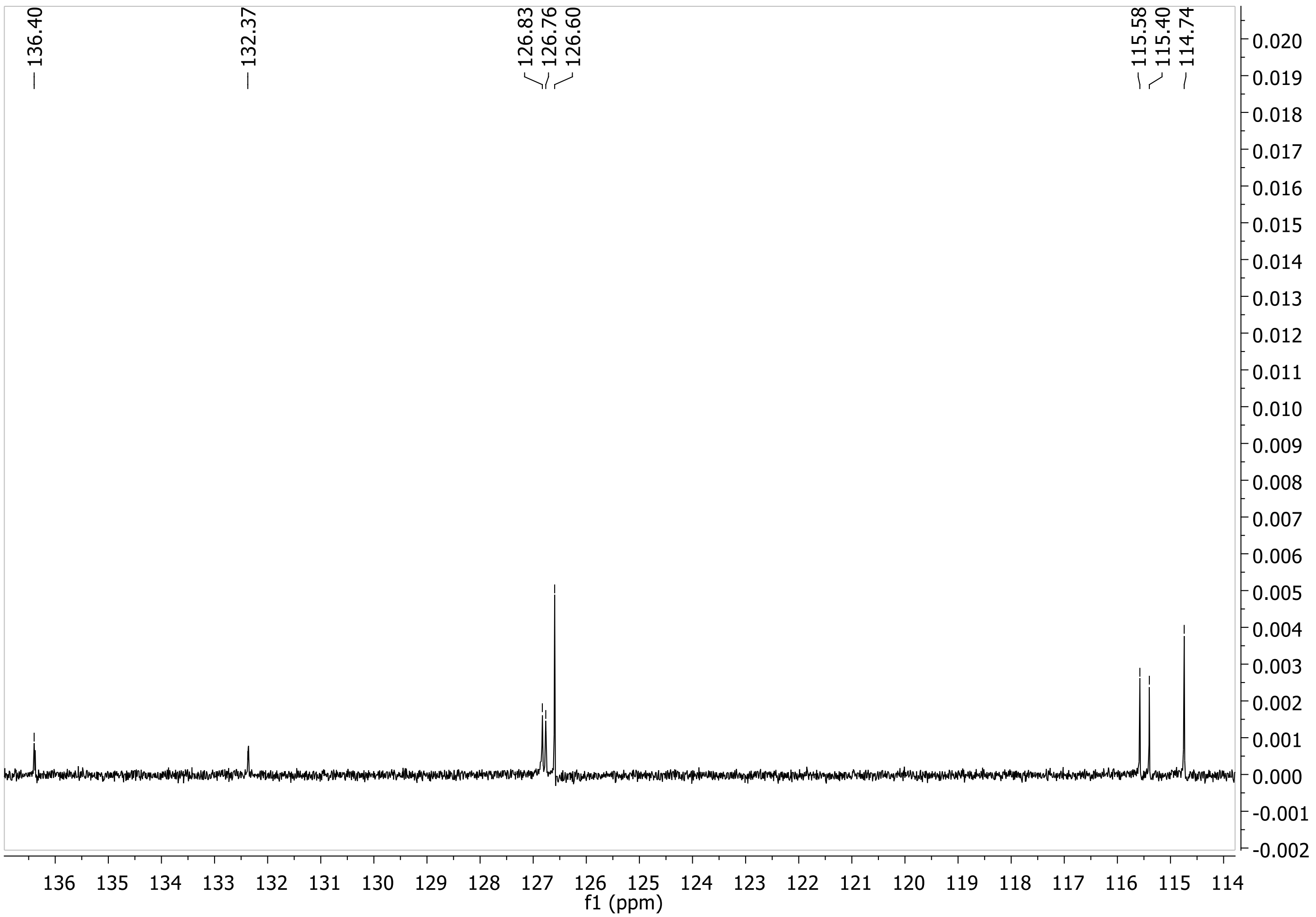

^{13}C NMR (DMSO-*d6*) of 1-(4-fluorophenyl)-3-(4-(hexyloxy)phenyl)thiourea **4**

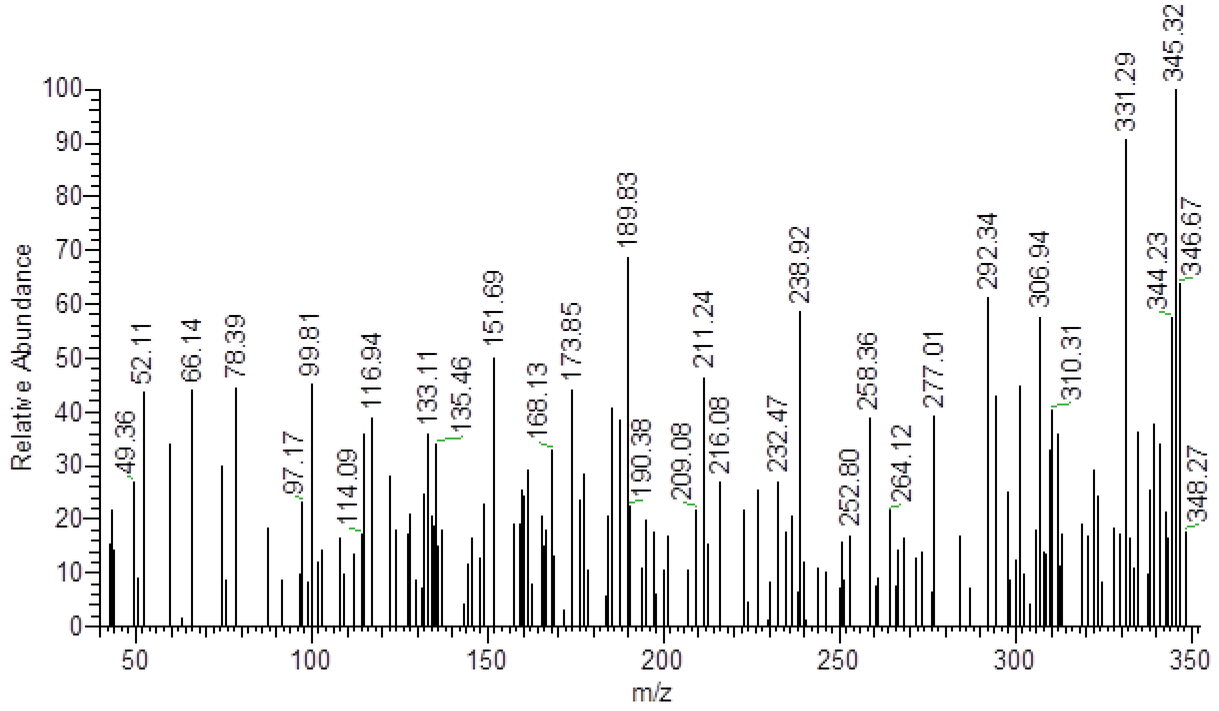

Mass spectrum of 1-(4-fluorophenyl)-3-(4-(hexyloxy)phenyl)thiourea **4**

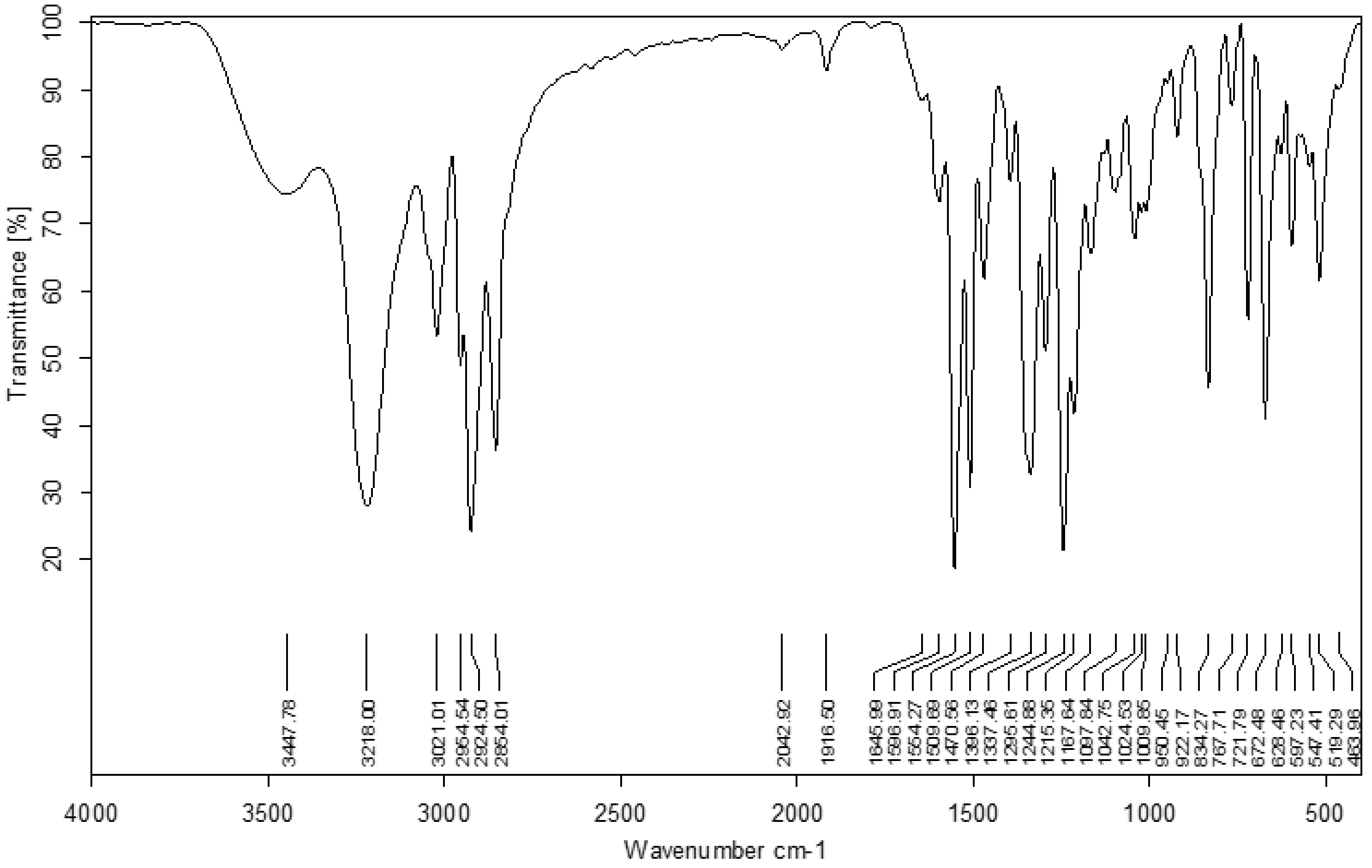

IR (KBr) spectrum of 1-(4-fluorophenyl)-3-(4-(octyloxy)phenyl)thiourea **5**

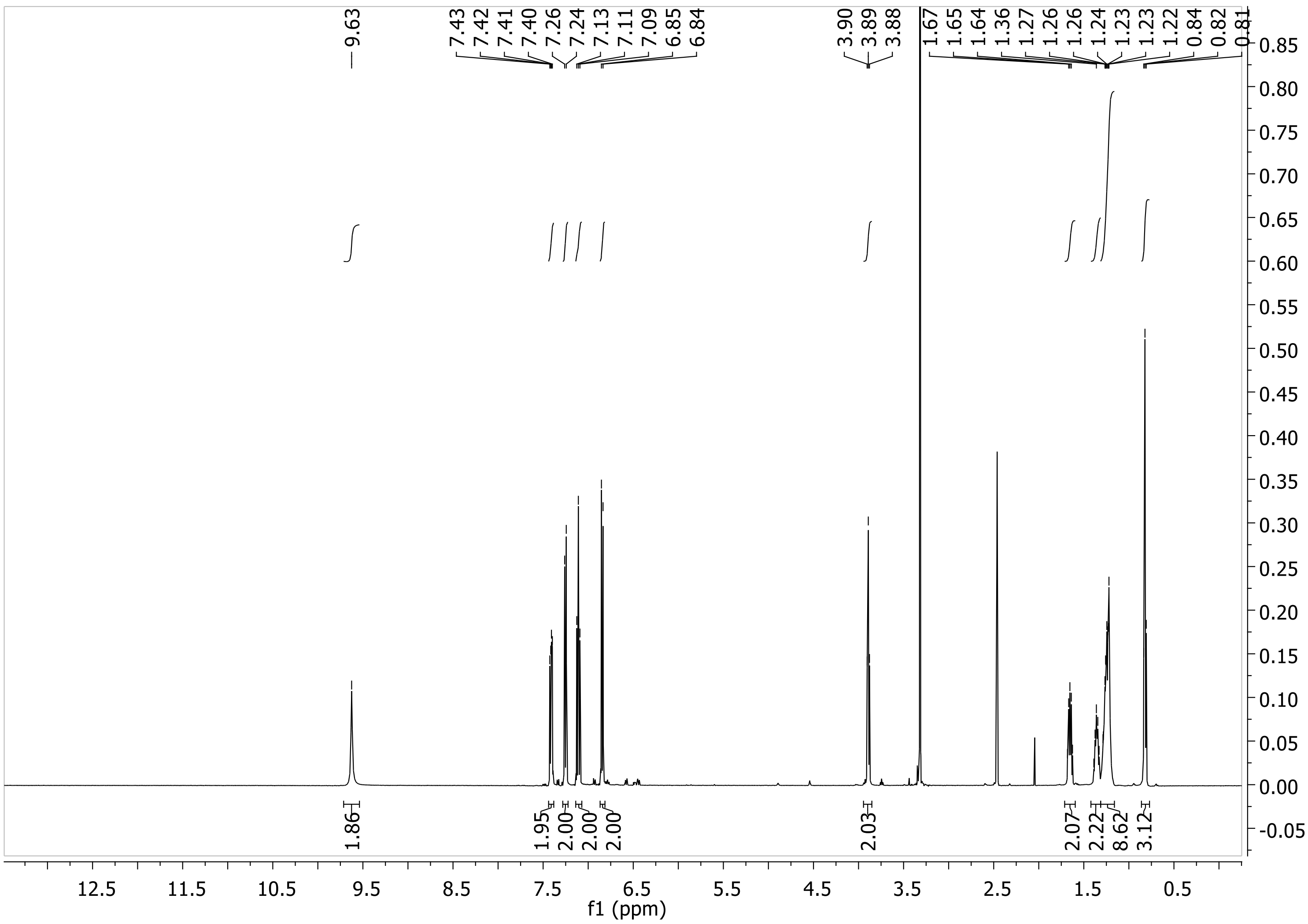

^{1}H NMR (DMSO-*d6*) spectrum of 1-(4-fluorophenyl)-3-(4-(octyloxy)phenyl)thiourea **5**

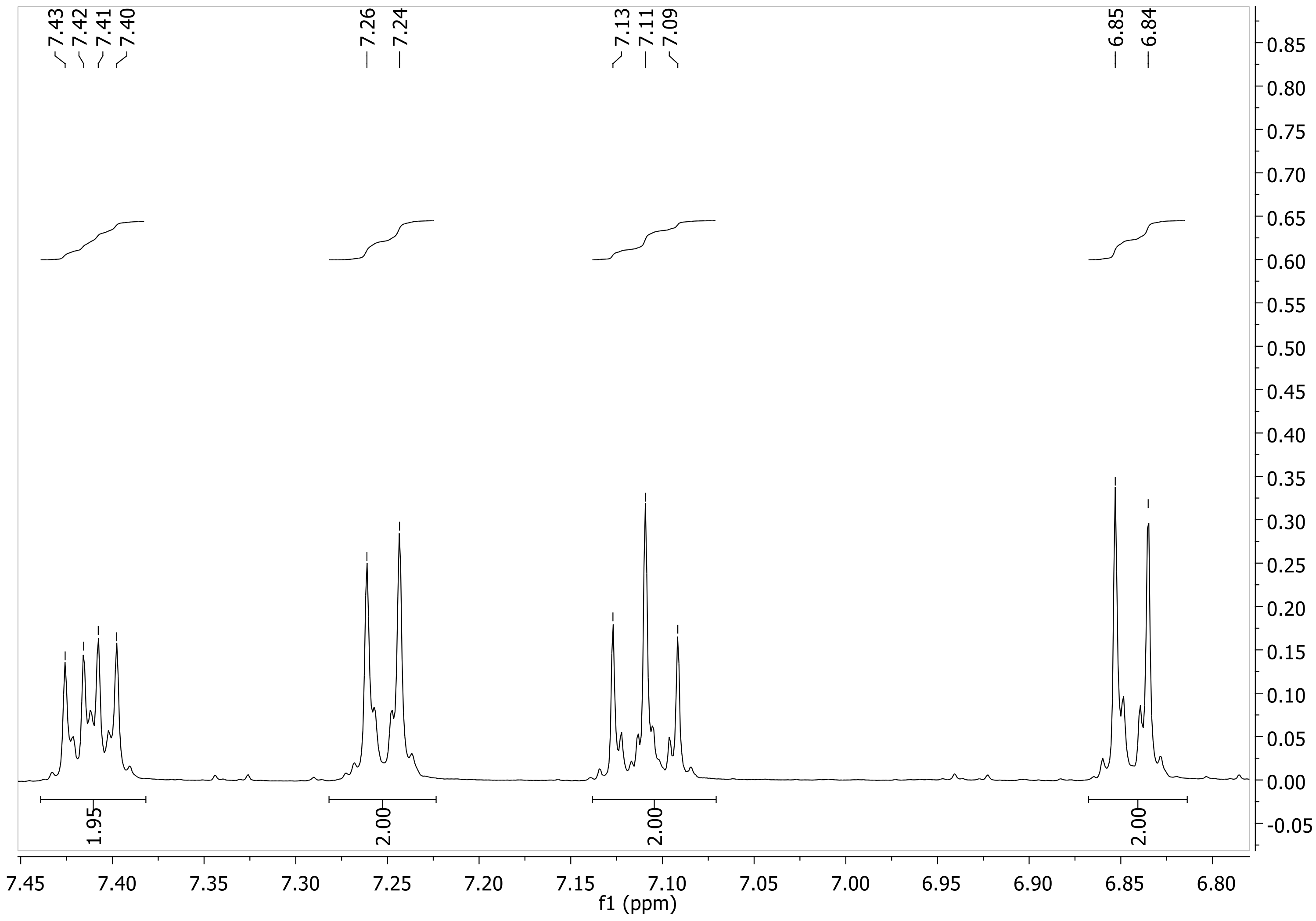

^{1}H NMR (DMSO-*d6*) spectrum of 1-(4-fluorophenyl)-3-(4-(octyloxy)phenyl)thiourea **5**

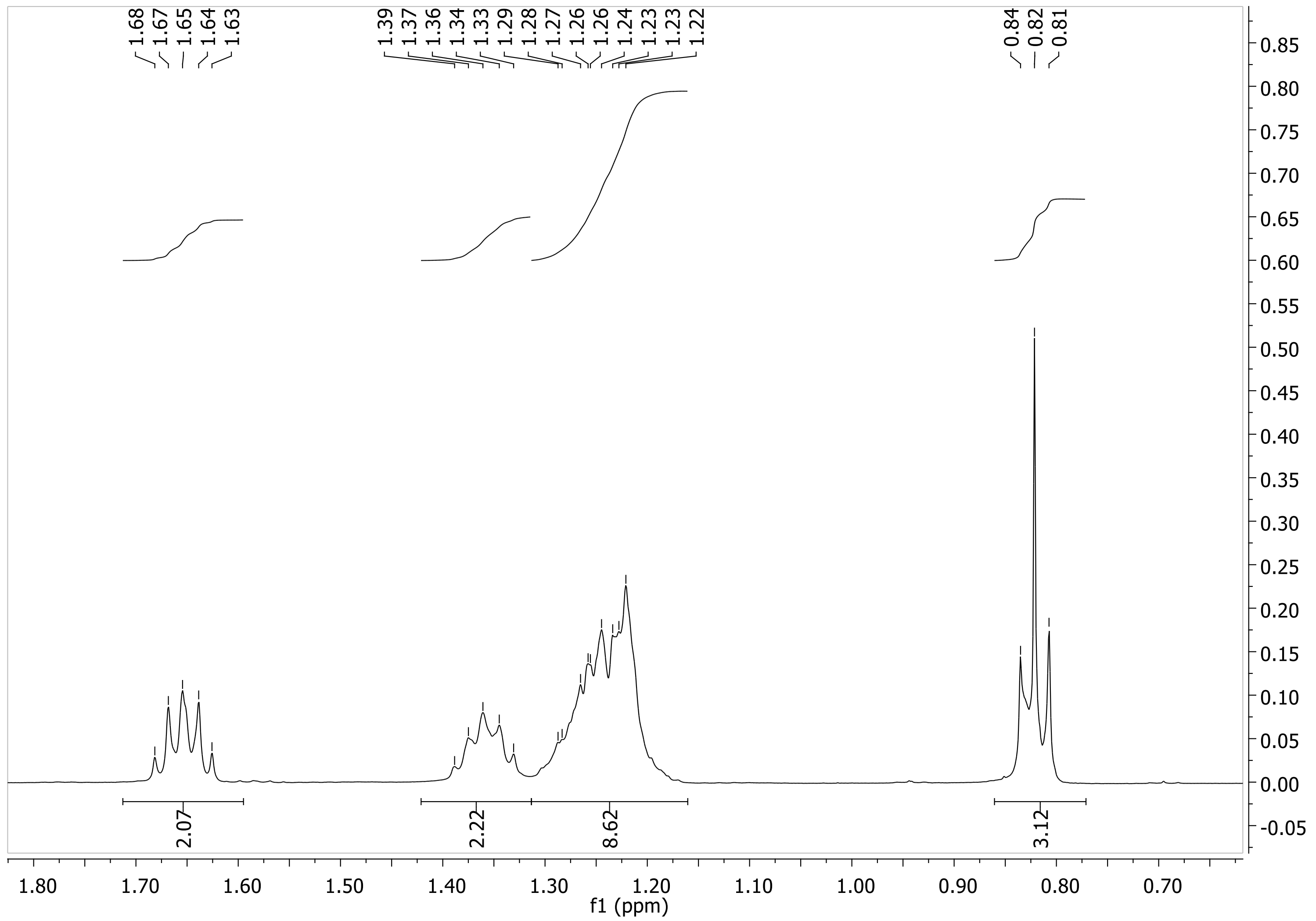

^{1}H NMR (DMSO-*d6*) spectrum of 1-(4-fluorophenyl)-3-(4-(octyloxy)phenyl)thiourea **5**

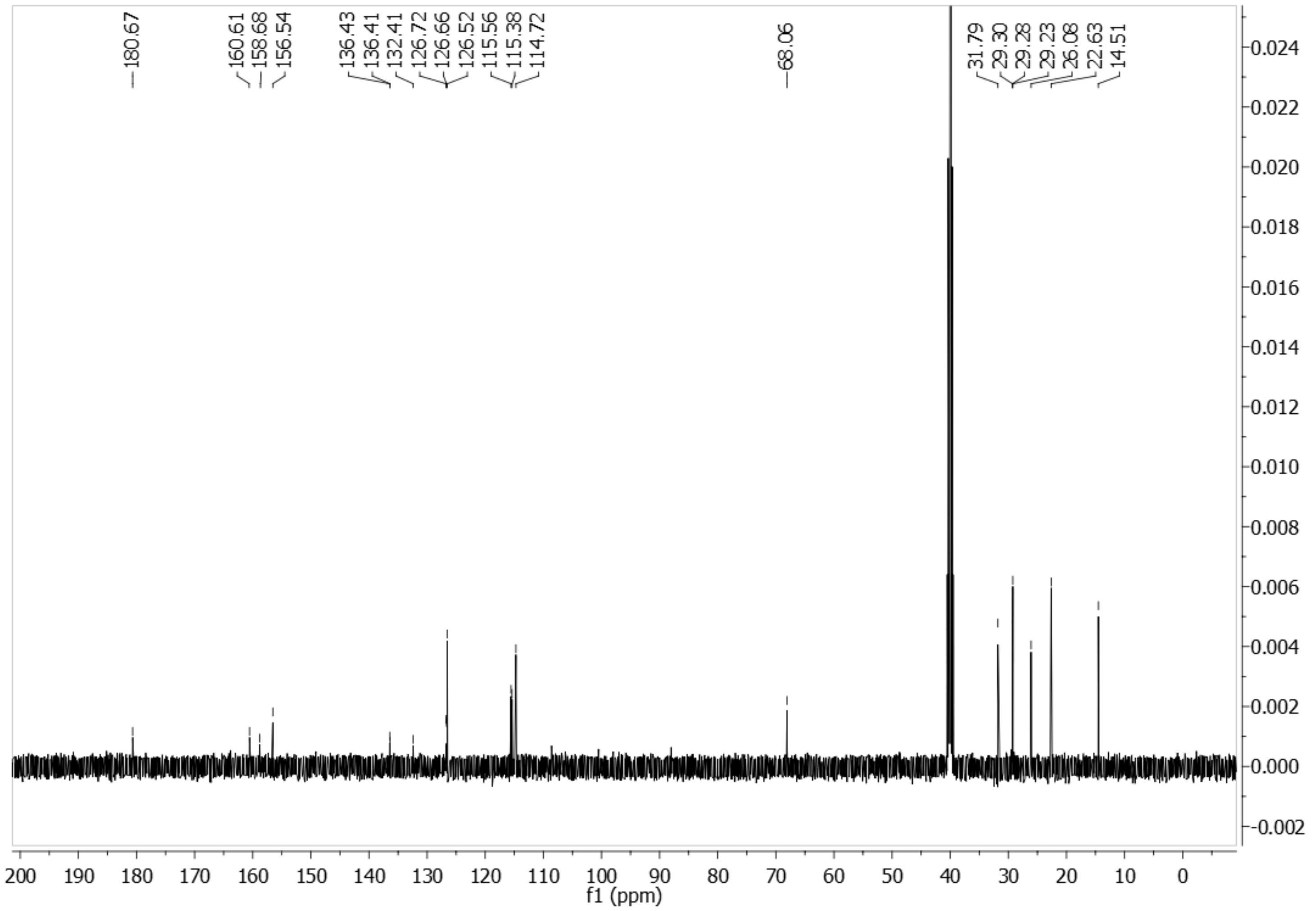

^{13}C NMR (DMSO-*d6*) of 1-(4-fluorophenyl)-3-(4-(octyloxy)phenyl)thiourea **5**

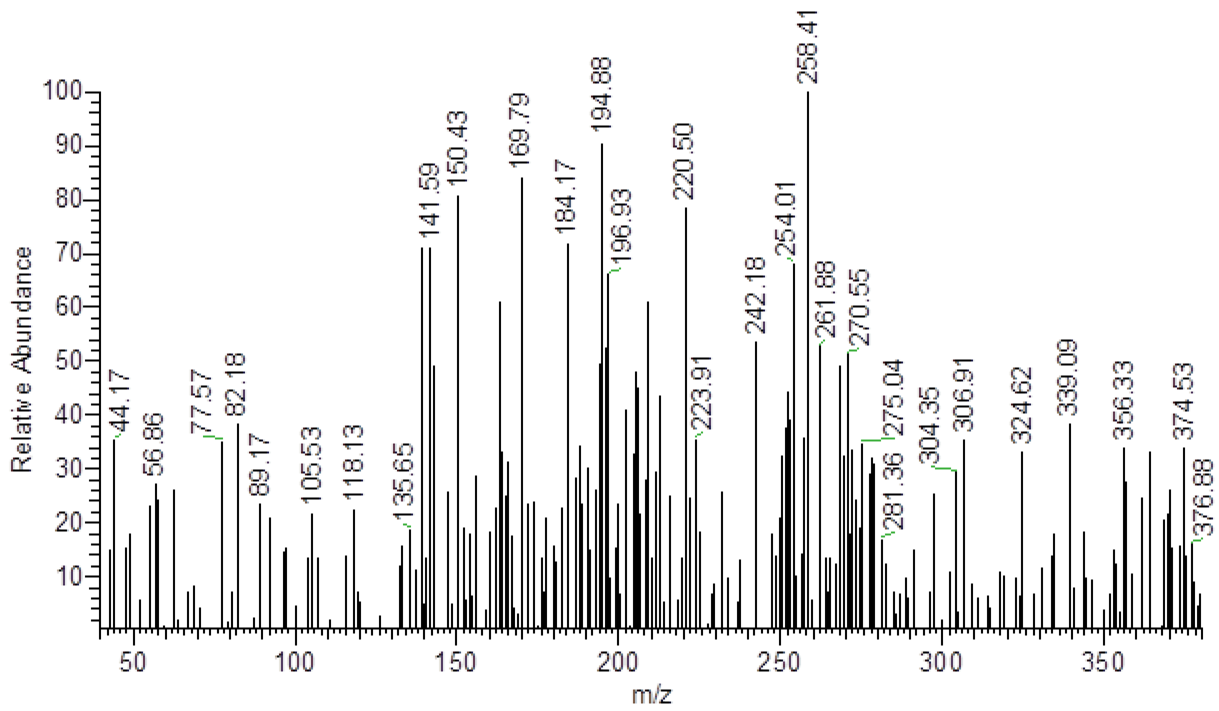

Mass spectrum of 1-(4-fluorophenyl)-3-(4-(octyloxy)phenyl)thiourea **5**

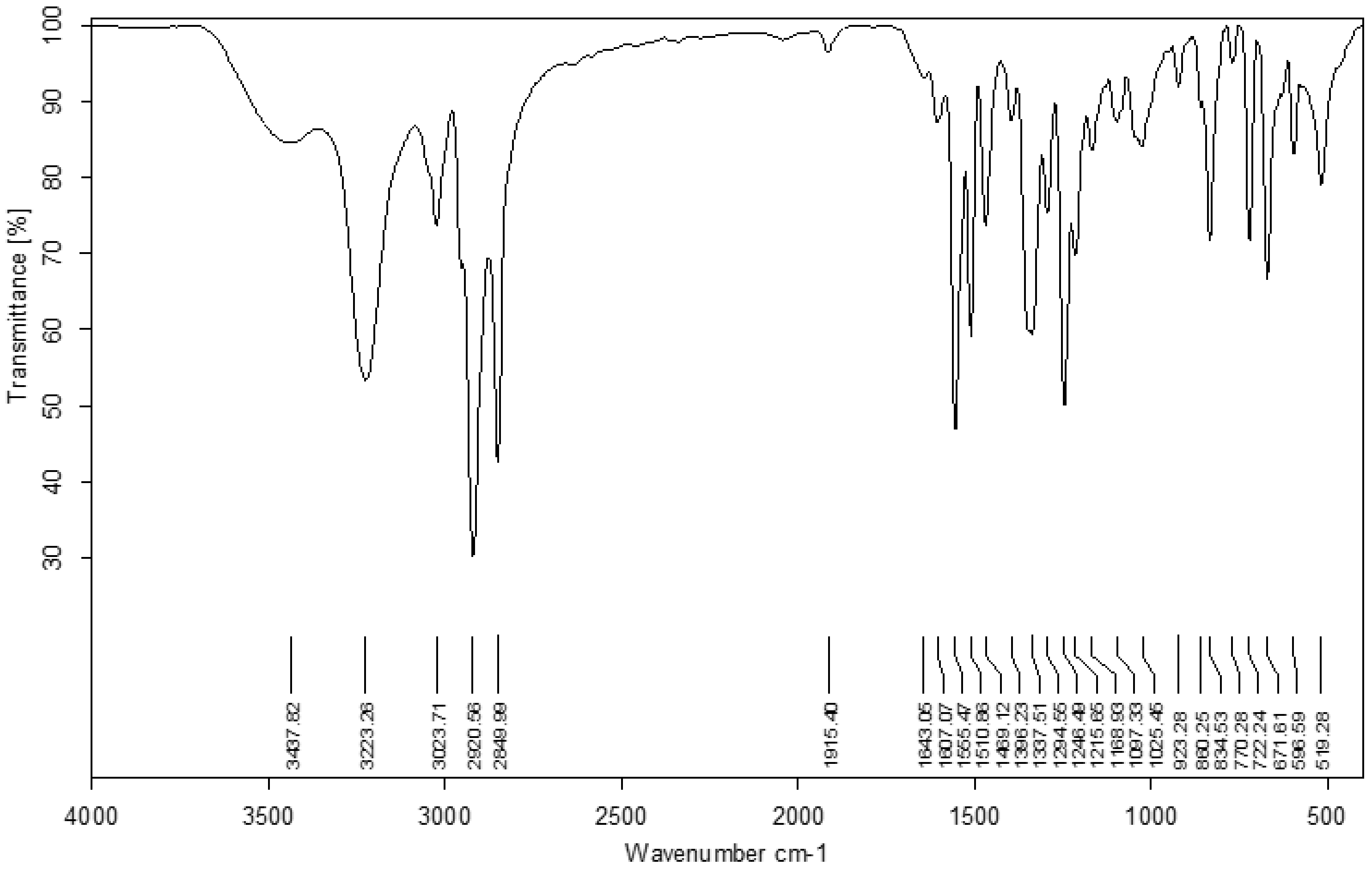

IR (KBr) spectrum of 1-(4-fluorophenyl)-3-(4-(hexadecyloxy)phenyl)thiourea **6**

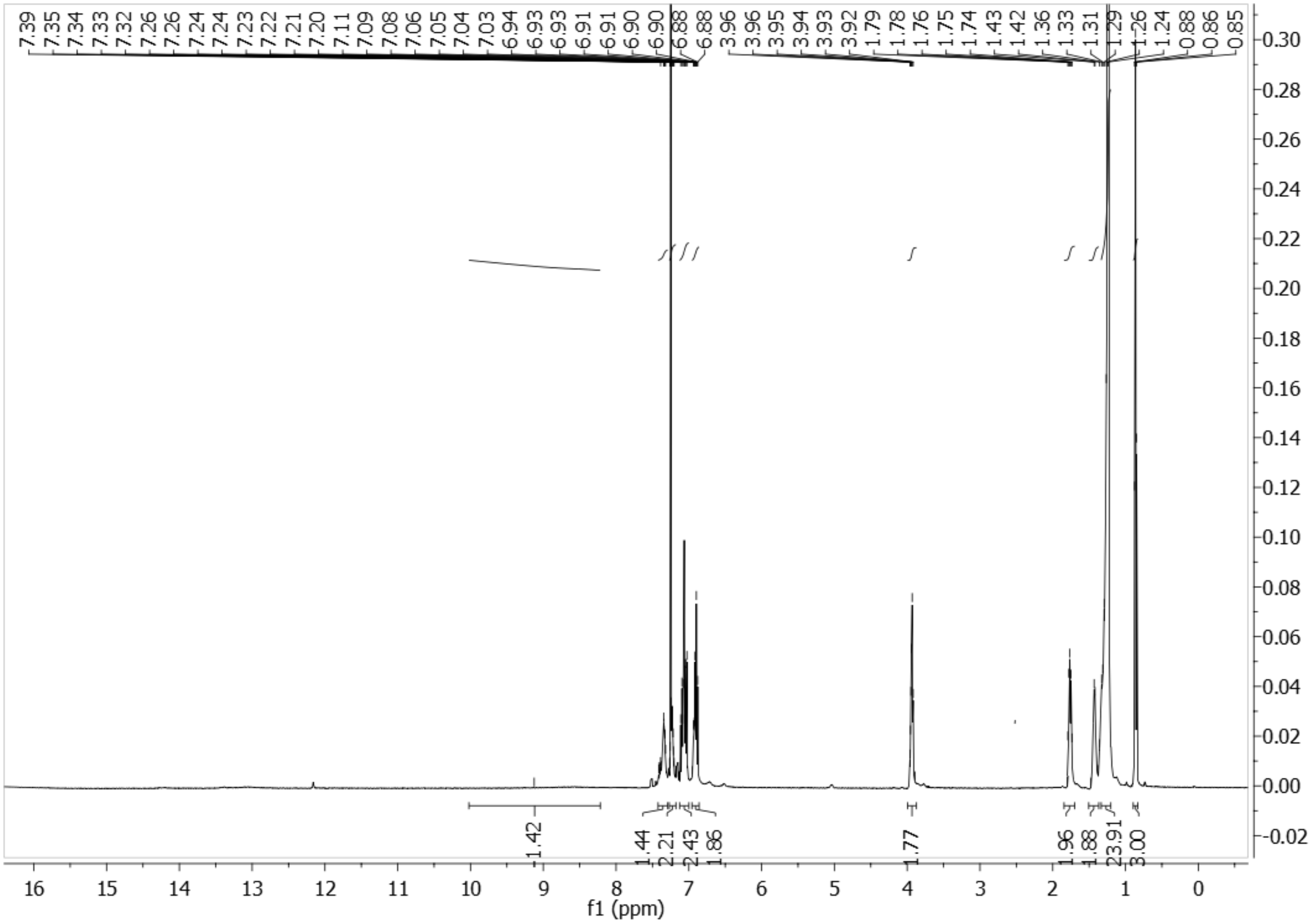

^{1}H NMR ($CDCl_3$) spectrum of 1-(4-fluorophenyl)-3-(4-(hexadecyloxy)phenyl)thiourea **6**

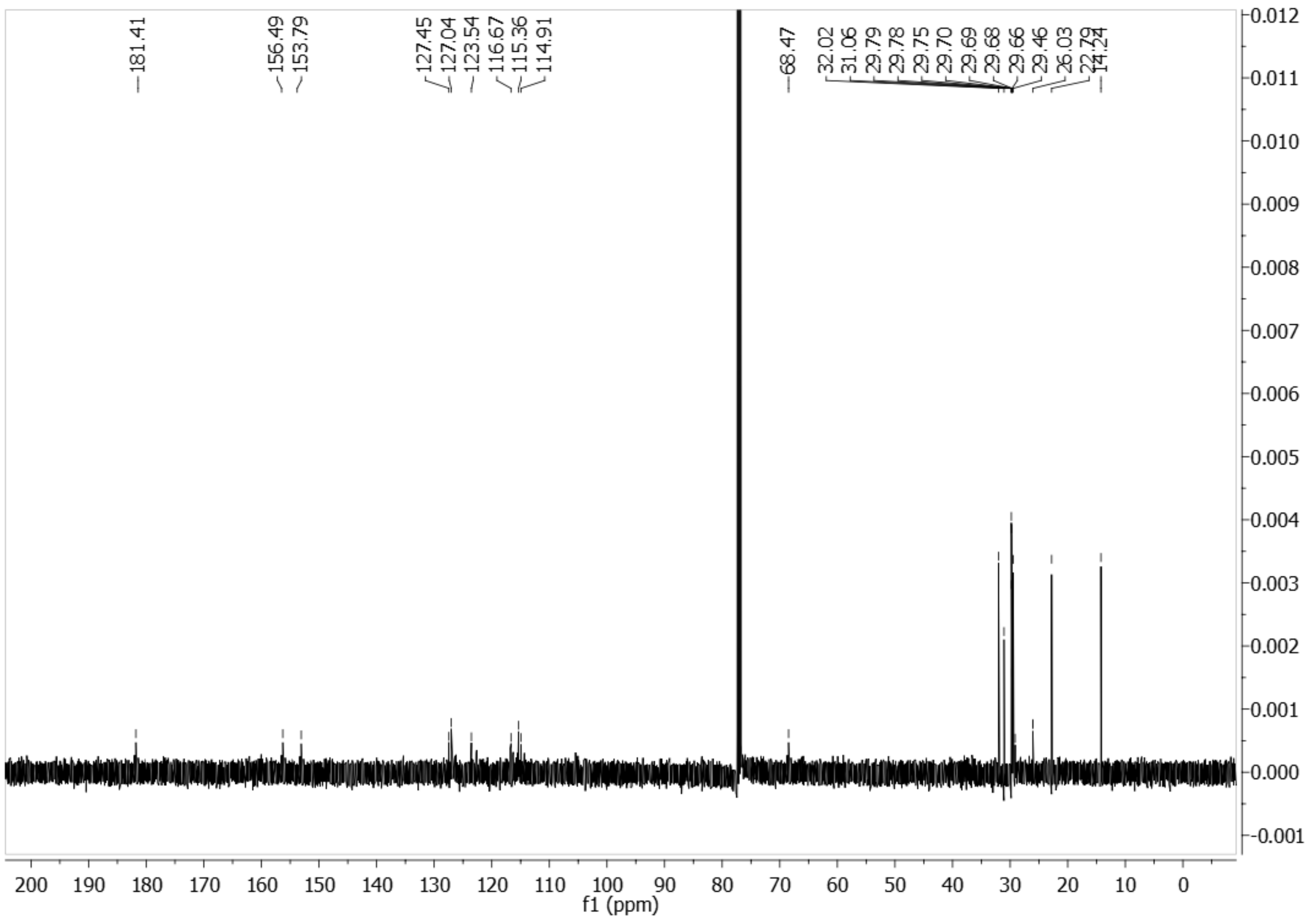

^{13}C NMR ($CDCl_3$) of 1-(4-fluorophenyl)-3-(4-(hexadecyloxy)phenyl)thiourea **6**

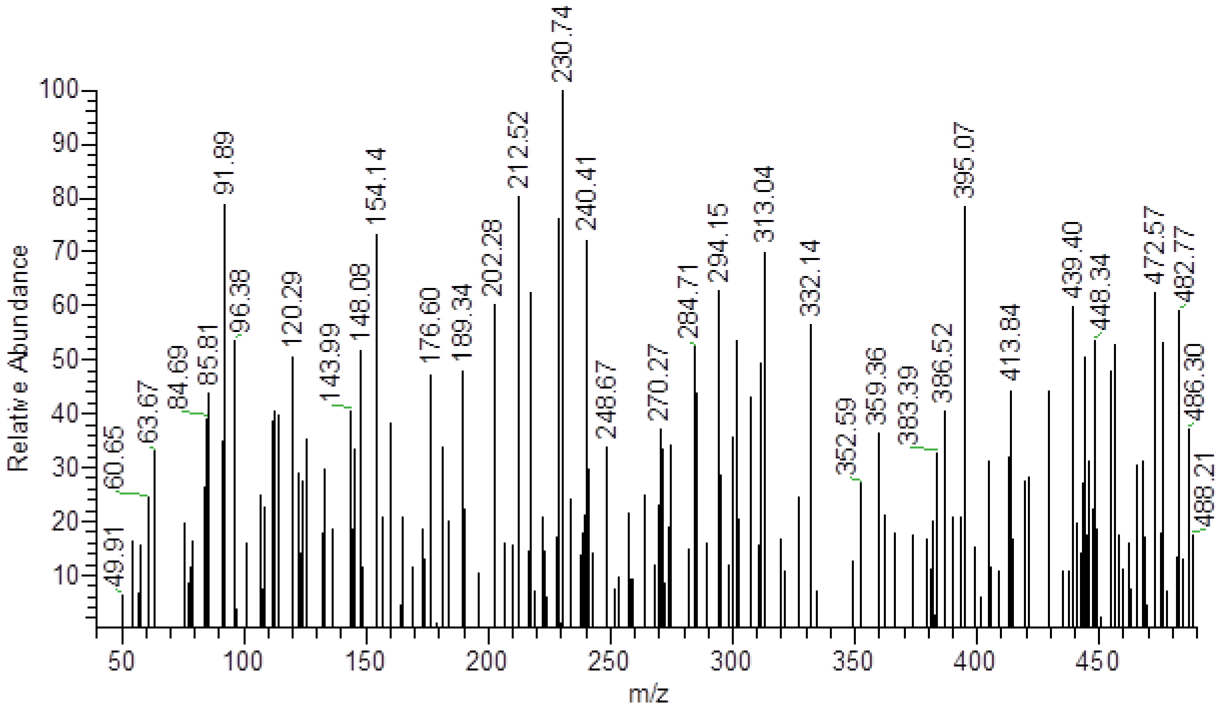

Mass spectrum of 1-(4-fluorophenyl)-3-(4-(hexadecyloxy)phenyl)thiourea **6**
